# A systematic review of calcium channel antagonists in bipolar disorder and some considerations for their future development

**DOI:** 10.1038/mp.2016.86

**Published:** 2016-05-31

**Authors:** A Cipriani, K Saunders, M-J Attenburrow, J Stefaniak, P Panchal, S Stockton, T A Lane, E M Tunbridge, J R Geddes, P J Harrison

**Affiliations:** 1 Department of Psychiatry, University of Oxford, Warneford Hospital, Oxford, UK; 2 Oxford Health NHS Foundation Trust, Warneford Hospital, Oxford, UK

## Abstract

l-type calcium channel (LTCC) antagonists have been used in bipolar disorder for over 30 years, without becoming an established therapeutic approach. Interest in this class of drugs has been rekindled by the discovery that LTCC genes are part of the genetic aetiology of bipolar disorder and related phenotypes. We have therefore conducted a systematic review of LTCC antagonists in the treatment and prophylaxis of bipolar disorder. We identified 23 eligible studies, with six randomised, double-blind, controlled clinical trials, all of which investigated verapamil in acute mania, and finding no evidence that it is effective. Data for other LTCC antagonists (diltiazem, nimodipine, nifedipine, methyoxyverapamil and isradipine) and for other phases of the illness are limited to observational studies, and therefore no robust conclusions can be drawn. Given the increasingly strong evidence for calcium signalling dysfunction in bipolar disorder, the therapeutic candidacy of this class of drugs has become stronger, and hence we also discuss issues relevant to their future development and evaluation. In particular, we consider how genetic, molecular and pharmacological data can be used to improve the selectivity, efficacy and tolerability of LTCC antagonists. We suggest that a renewed focus on LTCCs as targets, and the development of ‘brain-selective' LTCC ligands, could be one fruitful approach to innovative pharmacotherapy for bipolar disorder and related phenotypes.

## Introduction

Bipolar disorder is a common mental disorder with a lifetime prevalence of up to 4.4%.^1^ Mood stabilisation and prophylaxis is the principal aim of treatment. Despite the established efficacy of lithium and sodium valproate, manic and depressive episodes still recur in many patients, and all the existing drug treatments suffer from poor tolerability and potential harms.^2, 3^ There is a corresponding need for improved treatments.

Calcium signalling has long been implicated in bipolar disorder, following reports of altered levels of calcium in cerebrospinal fluid in patients with mania,^4, 5^ and the observation that long-term lithium treatment is associated with altered calcium metabolism, including hyperparathyroidism.^6^ These reports, taken together with the similarities in the mechanism of action of lithium and calcium channel blockers, prompted investigations of these drugs (primarily verapamil) beginning in the 1980s as potential treatments for bipolar disorder. This was facilitated by the fact that verapamil and other drugs that block l-type calcium channels (LTCC) were already available and in use for the treatment of hypertension and angina.^7, 8^ However, although studies reports have continued to emerge since that time regarding LTCC antagonists in bipolar disorder, the only evidence that has been systematically assessed concerns verapamil in the treatment of mania, with the data not demonstrating superiority over placebo.^9^

To investigate further the efficacy and tolerability of this class of drugs, we have conducted a systematic review of all LTCC antagonists in the treatment of acute episodes (both manic and depressive) and the prevention of relapse, in bipolar disorder. Our stimulus for doing so is that there is a renewed interest in the use of LTCC antagonists because the evidence for aberrant calcium signalling being important in the disorder has grown significantly in the past few years,^10, 11^ and LTCC antagonists are still mentioned in recent guidelines for the treatment of acute mania.^12^ The evidence is twofold. First, genomic data show that LTCC genes, especially *CACNA1C,* which encodes the Ca_v_1.2 alpha subunit,^13^ are part of the aetiology of bipolar disorder and several related phenotypes. Second, these genetic findings are complemented by new molecular and functional data arising from induced-pluripotent stem cell approaches, which considerably strengthen the prior evidence for aberrant calcium signalling in the pathophysiology of bipolar disorder and in the response to lithium therapy (see Discussion). Hence, in addition to a systematic review of the clinical data, we briefly review these recent findings and their implications for developing novel LTCC antagonists for use in bipolar disorder. Many of the considerations also apply to the potential role of this class of drugs for other neurological and psychiatric conditions such as Parkinson's disease and substance dependence.^14^

## Materials and methods

We followed the PRISMA guidelines^15^ and registered the review protocol on the PROSPERO website (http://www.crd.york.ac.uk/PROSPERO/display_record.asp?ID=CRD42015025465).

### Types of studies

We included randomised controlled trials (RCTs) comparing LTCC antagonists with placebo or any other active pharmacological treatment (all interventions could be in any preparation, dose, frequency, route of delivery or delivery setting). To assess efficacy and acceptability, we considered only double-blind studies. By contrast, for consideration of adverse effects, single blind or open RCTs were also included, and the most relevant non-randomised evidence was summarised as well. For RCTs with a crossover design, only results from the first period before crossover were considered. Cluster randomised trials were excluded. We included both published and unpublished studies. We allowed both fixed and flexible dose regimen designs. We excluded only studies recruiting participants with a serious concomitant medical illness.

### Types of participants

Patients of any age, of both sexes, of any ethnicity, based in any clinical setting, with a primary diagnosis of bipolar disorder (any subtype and according to any standardised diagnostic criteria) were included.

### Intervention

In addition to studies using LTCC antagonists as monotherapy, trials in which an LTCC antagonist was used as add-on treatment (for example, with lithium) were also included, if the pre-existing treatments were evenly distributed in both the experimental and comparator intervention arms, and were continued throughout the study. We only considered LTCC antagonists of the dihydropyridine, phenylalkylamine or benzothiazepine classes, as follows:

1. *Dihydropyridines:* amlodipine, aranidipine, azelnidipine, barnidipine, benidipine, cilnidipine, clevidipine, efonidipine, felodipine, isradipine, lacidipine, lercanidipine, manidipine, nicardipine, nifedipine, nilvadipine, nimodipine, nisoldipine, nitrendipine, pranidipine, ryodipine, trimetazidine.
2. *Phenylalkylamines:* anipamil, devapamil, falipamil, gallopamil, tiapamil, verapamil.
3. *Benzothiazepines:* clentiazem, diltiazem.

### Search strategy

Appropriate terms for bipolar disorder (bipolar disorder OR bipolar depression OR manic depression) and calcium channel blockers (using the terms listed in the previous section) were used. We searched the following electronic databases up to February 2016: the Cochrane Library, Medline, PreMedline, PubMed, EMBASE, CDSR, DARE, HTA, CINAHL and PsycINFO. International trial registries were searched for unpublished data (clinicaltrials.gov and the WHO registry for RCTs: http://www.who.int/ictrp/en/). No restrictions on date, language or publication status were applied. Appropriate journals and conference proceedings relating to bipolar disorder were hand-searched. Experts in this field were asked about any additional studies meeting the inclusion criteria of this systematic review. Full details on the search strategy are reported in Supplementary Appendix 1.

### Outcome measures

#### Acute treatment

The main outcomes for the efficacy of LTCC antagonism in the treatment of acute mood episodes were (i) hospital admission during the study period, (ii) length of hospital admission, (iii) time to cessation of additional treatment for manic/depressive symptoms, (iv) changes on validated manic/depressive symptom rating scales from baseline, (v) changes on validated psychotic symptom rating scales from baseline and (vi) response to treatment, defined as showing an improvement of at least 50% on any validated mania/depression rating scale.

#### Long-term treatment

The main outcomes for the efficacy of LTCC antagonists in the long-term treatment of bipolar disorder were (i) time to recurrence of any mood episodes, (ii) number of recurrences of any mood episodes during the trial period, (iii) number of recurrences of manic episodes during the trial period, (iv) number of recurrences of mixed episodes during the trial period and (v) number of recurrences of depressive episodes during the trial period. Recurrence was defined either as (i) study withdrawal due to recurrence of any mood episode, (ii) admission to hospital (time to next admission and number of admissions during trial period), or (iii) institution of additional treatment for any mood episode and time to institution.

### Assessment of risk of bias in included studies

Two review authors (KS, MJA) independently assessed risk of bias for each study included in the efficacy and acceptability analyses, using the criteria outlined in the Cochrane Handbook for Systematic Reviews of Interventions.^16^ Any disagreements were resolved by discussion or by involving another review author (AC). We assessed the risk of bias according to the following domains: (i) random sequence generation, (ii) allocation concealment, (iii) blinding of participants and personnel, (iv) blinding of outcome assessment, (v) incomplete outcome data, (vi) selective outcome reporting, (vii) other sources of bias. We judged each potential source of bias as high, low or unclear, and provide a supporting quotation from the study report together with a justification for our judgement in the ‘Risk of bias' table (Supplementary Appendix 2). We summarised the risk of bias judgements across different studies for each of the domains listed. We considered blinding separately for different key outcomes where necessary (for example, for un-blinded outcome assessment, risk of bias for all-cause mortality may be very different than for a participant-reported mood scale).

### Data collection and statistical analysis

At least two researchers from the review team (MJA, JS, KS, PP and AC) independently identified eligible studies. For dichotomous data, the risk ratio was calculated with its 95% confidence interval (CI). For statistically significant results, we planned to calculate the number needed to treat for an additional beneficial outcome and the number needed to treat for an additional harmful outcome as the inverse of the risk difference. For continuous data, mean differences (MDs) or standardised mean differences (SMDs) were calculated with 95% CIs. MDs were used when the same scale was used to measure an outcome, whereas SMDs were employed when different scales were used to measure the same outcome. For multi-arm studies, we considered whether each possible pair-wise comparison of interventions in the study was eligible for the meta-analysis. Binary outcomes were calculated on a strict intention-to-treat basis as dropouts were always included in the analyses. When data were missing and the method of ‘last observation carried forward' used to do an intention-to-treat analysis, then the last observation carried forward data were used. When standard deviations (SDs) were not reported, we asked authors to supply the data. When only the standard error or *t*-statistics or the *P* value was reported, we calculated SDs in accordance with Altman and Bland.^17^ Heterogeneity between studies was investigated by the *I*^2^ statistic^18^ and by visual inspection of the forest plots. We used the Cochrane Handbook for Systematic Reviews of Interventions' rough guide to its interpretation as follows: 0–40% might not be important; 30–60% may represent moderate heterogeneity; 50–90% may represent substantial heterogeneity; and 75–100%, considerable heterogeneity. We also kept in mind that the importance of the observed value of *I*^2^ depends on (i) the magnitude and direction of effects and (ii) the strength of evidence for heterogeneity (for example *P* value from the χ^2^ test, or a CI for *I*^2^). If the *I*^2^ value was below 50%, but the direction and magnitude of treatment effects were suggestive of important heterogeneity, we investigated the potential sources of heterogeneity. We reported *I*^2^ values in all analyses including two or more studies. One sensitivity analysis was planned, excluding trials in which LTCC antagonists were used as add-on treatment, in order to determine if co-prescription may affect the efficacy of the investigational drug.

## Results

**Figure 1:**
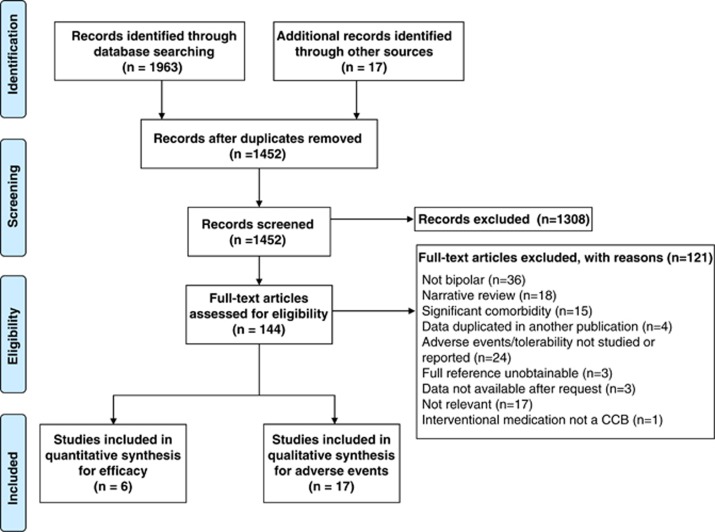
PRISMA flow chart with included and excluded studies, with reasons.^15^

The electronic searches revealed 1453 potentially relevant studies (Figure 1). Following review of titles and abstracts, 144 potentially eligible studies were identified. We excluded 121 that did not meet the eligibility criteria. Seventeen studies were identified from searching the trial registers. In total 23 studies published between 1984 and 2014 contributed with usable data and were included in the review.^19, 20, 21, 22, 23, 24, 25, 26, 27, 28, 29, 30, 31, 32, 33, 34, 35, 36, 37, 38, 39, 40, 41^ The great majority of the outcomes of interest prespecified in the protocol could not be analysed because of the lack of available data for these outcomes. We extracted all usable data from the included studies and contacted the authors of the randomised trials, if necessary.

### Double-blind RCTs

**Figure 2:**
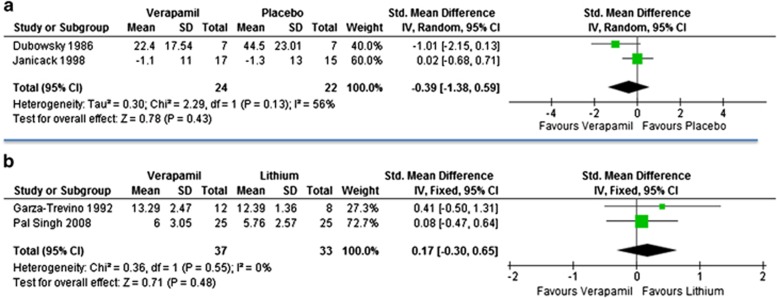
Forest plots with efficacy as severity of symptoms (SMD) in acute mania: verapamil versus placebo (**a**) and verapamil versus lithium (**b**). CI, confidence interval; SD, standard deviation; SMD, standardised mean difference.

Six double-blind randomised studies^19, 20, 21, 22, 23, 24^ were identified (Table 1). All six studies compared verapamil with either placebo (two trials) or lithium (four trials) in people with acute mania; one study recruited lithium-resistant patients.^23^ Of the six RCTs, four recruited only inpatients, one included inpatients and outpatients^23^ and in one study this information was unclear.^21^ In total 81 patients were randomly assigned to receive verapamil and 76 received other compounds (placebo=22, lithium=54). The meta-analysis indicated that verapamil was not superior to placebo (SMD −0.39, 95% CI: −1.38 to 0.59) and lithium was not statistically significantly better than verapamil (SMD 0.17, 95% CI: −0.30 to 0.65) in the treatment of manic symptoms (Figure 2). The study that recruited only lithium-resistant patients found no difference between lithium and verapamil in terms of number of people who responded to treatment (4 out of 8 for lithium versus 3 out of 10 for verapamil; risk ratio 0.60, 95% CI: 0.19 to 1.94). In terms of dropout rate, placebo resulted with more patients terminating the study (6 out of 15 versus 3 out of 17; risk ratio 2.27, 95% CI: 0.68 to 7.52), however the difference was not statistically significant. It was not possible to analyse the acceptability data for lithium and verapamil, because of the two studies that reported withdrawal rate, one did not have dropouts^24^ and the other one did not report the actual number of patients originally randomised.^20^ Two studies^20, 24^ reported data about adverse events, but no significant differences were found between verapamil and lithium (data not shown, available from the authors). A summary of the overall risk of bias is presented in Supplementary Appendix 2.

### Observational studies

We found 17 observational studies that were included for the consideration of adverse events only: two non-randomised double-blind trials,^25, 26^ seven open label studies^27, 28, 29, 30, 31, 32, 33^ and eight case reports.^34, 35, 36, 37, 38, 39, 40, 41^ Verapamil was the most commonly used LTCC antagonist (*N*=11) with two studies using diltiazem, and single studies using nimodipine, nifedipine, methyoxyverapamil or isradipine. Full description of the characteristics of these studies is reported in Supplementary Appendix 4.

Verapamil was associated with headache,^29, 32, 33, 34, 39, 41^ and changes in blood pressure and heart rate. ^26, 30, 31, 34, 40^ There was one case report of sinus bradycardia with atrioventricular ectopy where the patient died 3 days later of a myocardial infarct.^42^ Toxic delirium was reported in one study,^37^ which resolved on withdrawal of verapamil. Two studies reported the emergence of ataxia,^35, 36^ and a further study reported a patient who developed involuntary choreoathetoid movements following treatment with verapamil;^38^ in all three cases the symptoms resolved on withdrawal of the drug. Diltiazem was reported to be associated with headache, vertigo, peripheral oedema and nausea.^29, 32^ Nifedipine was reported to cause similar side effects although the majority of these occurred in the same four patients (a group that had also experienced these problem with lithium) and were deemed by the authors to be a particularly sensitive subgroup.^33^ Sleep disturbance was the only side effect noted in those taking nimodipine, although this seems likely to relate to the drug being administered at 4 hourly intervals through the night.^28^ Isradipine was reported to have caused transient decreases in diastolic blood pressure and increases in heart rate, which had resolved after 4 weeks of treatment.^31^

A summary of all studies identified where LTCC antagonists have been used in bipolar disorder, including those ineligible for inclusion in this systematic review, is given in Supplementary Appendix 4.

## Discussion

To the best of our knowledge, this is the first systematic review and meta-analysis of LTCC antagonists in all phases of bipolar disorder. We found six RCTs compared to the only previous systematic review including LTCC antagonist studies in the treatment of bipolar disorder,^9^ which found just one. Notwithstanding the comprehensive search and the inclusion of unpublished data, the small number and low quality of double-blind RCTs highlights the limited evidence and consequent substantial residual clinical uncertainty about the efficacy and acceptability of LTCC antagonists in bipolar disorder. Our findings agree with the conclusion of Yildiz *et al.*^9^ that verapamil has not been demonstrated to be efficacious for the treatment of mania, and show that no conclusions can be drawn regarding the other LTCC antagonists in mania. No controlled data were identified for LTCC antagonists in the prophylaxis of bipolar disorder, nor for bipolar depression. However, for the latter indication, a preliminary open study suggested that isradipine may be efficacious,^31^ and there is a currently ongoing double-blind, placebo-controlled RCT (https://clinicaltrials.gov/ct2/show/NCT01784666).

Adverse events were poorly reported across all study types, with the commonly reported side effects being all related to the peripheral actions of LTCC antagonists and being predominantly cardiovascular in nature. The tolerability of LTCC antagonists in bipolar disorder is therefore unclear, although they have proved to have a reasonable side effect profile when used for hypertension or angina.^43, 44^ However, there are several safety concerns, which may impact on their use in bipolar disorder, especially if long-term use were envisaged. First, a large population-based case–control study linked LTCC antagonists with breast cancer.^45^ Although larger case–control and cohort studies have failed to find any association for all cancers or breast cancer,^46, 47^ a recent meta-analysis of almost 150 000 subjects reported an association between long-term LTCC antagonist treatment and breast cancer (risk ratio=1.71).^48^ Second, it has been suggested that LTCC antagonists are associated with increased suicide risk.^49^ However, this study did not control important independent risk factors such as depression, did not validate diagnoses of suicide and the finding was not replicated in a British case–control study^50^ nor in a large Danish case–control study.^51^ Third, currently available LTCC antagonists (particularly immediate release preparations) are potentially lethal in overdose, largely as a result of their cardiovascular effects. Fourth, some early case reports described depression occurring during treatment with LTCC antagonists.^52^ Finally, there is the potential for LTCC antagonists to interact with other drugs commonly prescribed in bipolar disorder. LTCC antagonists may enhance lithium excretion and may have synergistic neurotoxicity.^7, 36^ Both verapamil and diltiazem increase carbamazepine levels, and neurotoxicity has been reported.^53, 54^

Our systematic review has several limitations. Overall, the quality of included studies was poor and risk of bias across studies was assessed as ‘unclear' for the majority of domains, because most studies did not provide enough information to enable a risk of bias assessment. This restricts the interpretation and reliability of these results. The assessment of bias was based on the adequacy of blinding attempts as described in each paper's methods, not on the actual degree of blinding achieved. We rated studies as ‘low risk' when all measures used to blind study participants and personnel from knowledge of which intervention a participant received was described. We rated studies as ‘unclear risk' when there was a lack of information on blinding procedures. Of the six included studies assessing the efficacy of LTCC antagonists, none tested the blind or provided any information relating to whether the intended blinding was effective. Even though we carried out an extensive search of all the published and unpublished literature available to us, we cannot rule out the possibility that some relevant information was missed out during the review process. The main limitation of our systematic review is, however, that the lack of high-quality data from randomised trials means that no clear conclusions can be drawn regarding the efficacy of LTCC antagonists in bipolar disorder, more than 30 years after their use was first described.

## Looking ahead: towards bespoke LTCC antagonists for bipolar disorder and other psychiatric indications

At first sight, the results of this systematic review suggest that LTCC antagonists hold no or only very little promise for the treatment of bipolar disorder, and that novel therapeutic efforts should be directed elsewhere. However, we consider this would be premature for two reasons, in addition to the paucity of existing RCT data. First, the scientific rationale for targeting calcium channels in bipolar disorder has strengthened significantly in the past few years. Second, there are grounds for believing that the existing studies considerably underestimate the potential value of this class of drugs (Table 2).

### LTCCs in bipolar disorder and its treatment

As noted earlier, there is long-standing evidence for calcium dysregulation in bipolar disorder and for lithium-induced effects on calcium dynamics.^55, 56^ This evidence has recently been complemented by demonstration of altered calcium channel gene expression and calcium signalling in neurons derived from patients with bipolar disorder compared to control subjects, and from lithium-responsive patients compared to lithium non-responders.^57, 58, 59, 60, 61^ For example, Hahn *et al.*^57^ showed lower basal and stimulated intracellular calcium levels in olfactory receptor neurons from unmedicated euthymic bipolar patients compared to controls, whereas Chen *et al*^58^ reported increased expression of calcium signalling-related transcripts in induced-pluripotent stem cell-derived neurons from bipolar disorder patients. A similar finding was also described recently by Mertens *et al*,^59^ who also reported that the cellular phenotype was normalised by lithium in patients who had responded clinically to the drug, but not in those who had not responded.^61^

A causal role for altered calcium signalling in bipolar disorder, mediated via LTCCs, is now apparent from recent genetic studies. *CACNA1C*, which encodes the LTCC Ca_v_1.2 α_1_ subunit, is most robustly linked; single nucleotide polymorphisms at this locus show association with bipolar disorder in genome-wide association studies^62, 63, 64^ and rare variants are linked with bipolar disorder in multiply-affected families.^65^ *CACNA1C* is further implicated by whole-genome sequencing of bipolar disorder patients and controls,^66^ and by its altered expression in the frontal cortex of patients with bipolar disorder.^67^ These findings together increase the rationale for therapeutic targeting of LTCCs, in particular Ca_v_1.2. Although the mechanism of disease association is not known, the balance of evidence suggests that *CACNA1C* risk single nucleotide polymorphisms are associated with increased LTCC expression and function.^11, 68, 69^ In particular, induced neurons from subjects with the risk *CACNA1C* single nucleotide polymorphism show greater expression of calcium channel subunit mRNA, and increased calcium signalling, compared to those without the risk allele.^69^ This is consistent with the bulk of the earlier biochemical evidence in bipolar disorder, which indicates enhanced calcium signalling and supporting the assumption that antagonism of the channels would be therapeutically desirable.

Associations between LTCC genes and psychiatric illness are not limited to *CACNA1C*, nor to bipolar disorder. There is also genome-wide significant association of bipolar disorder with *CACNA1D* (which encodes the Ca_v_1.3 α1 subunit) and *CACNB3* (which encodes the β3 subunit) loci^70^ and studies of rare variants suggest involvement of several other LTCC subunits, including *CACNA1D*.^65^ *CACNA1C* is also a risk locus for major depression and schizophrenia,^70, 71^ while the *CACNB2* locus confers susceptibility to multiple psychiatric disorders including bipolar disorder.^70^ As well as these diagnostic associations, *CACNA1C* and other LTCC subunits are part of the genetic contribution to cognition and sleep. Thus, *CACNA1C* shows genome-wide significance with working memory performance,^72^ whereas other LTCC alpha subunits contribute to episodic memory.^73^ *CACNA1C* genotype also impacts on memory-related brain activity^67, 74, 75^ and functional connectivity.^76^ Regarding sleep, variants in *CACNA1C* have been associated by genome-wide association study with sleep latency^77^ and sleep quality.^78^ Although these latter findings need replication, they highlight a likely role for LTCCs in memory and circadian rhythms, both of which are important features of bipolar disorder, and are thus potential targets for treatment using LTCC antagonists.^79, 80^ That is, LTCC antagonists may have value in normalising sleep, or improving cognition in bipolar disorder, as well as or in place of a primary effect on treating or stabilising mood. *CACNA1C* genotype may also influence other domains relevant to bipolar disorder and its therapy, including resilience, depressive symptoms and reward responsiveness, but these require further investigation.^81, 82^

Additional support for the therapeutic candidacy of LTCCs for bipolar disorder and other psychiatric indications comes from recent studies into their distribution and function. LTCC identity is determined by the α1 subunit, which forms the Ca^2+^-selective pore and contains the voltage sensor and most regulatory binding sites, whereas LTCC function (for example, trafficking) is regulated by accessory subunits, including the β-subunits.^83, 84^ Of the alpha subunits, Ca_v_1.2 and Ca_v_1.3 are the predominant subunits expressed in neurons^83, 85, 86^ where they are located postsynaptically in dendritic spines and shafts.^87^ They are involved in dendritic signalling^88, 5^ and have an important role in signalling from the synapse to the nucleus (‘excitation-transcription coupling'), which is important for hippocampal long-term potentiation, one of the key processes underlying memory.

In summary, recent genomic, molecular and pharmacological findings provide convergent evidence that LTCCs are an important player in the pathophysiological mechanisms underlying bipolar disorder and some of its component phenotypes (memory and sleep). The case for therapeutically targeting these channels is correspondingly strengthened. In addition, trials using LTCC antagonists in bipolar disorder can (and should) now select or stratify participants based on *CACNA1C* risk genotype, as this may modify the treatment effect,^31, 69^ and in due course may allow pharmacogenetic prediction of response.

### Pharmacological and other considerations for LTCC antagonist drug discovery

Despite the enhanced rationale for LTCC antagonism provided by the recent data, there remain other major issues, which need to be taken into account if the therapeutic potential of this approach is to be realised. It is clear that, for various reasons, the existing licensed LTCC antagonists are unlikely to provide sufficient potency, tolerability and safety. For example, the older drugs (notably verapamil and diltiazem) have either poor or uncertain blood–brain barrier permeability and hence may not produce sufficient channel blockade for efficacy in bipolar disorder or any other disorder requiring central nervous system LTCC occupancy.^89, 90, 91, 92^ Whereas this is less of a problem for most of the newer drugs,^93, 94, 95^ they all suffer from one or more other limitations. In particular, they all block LTCCs located in the heart and vasculature at least as effectively as they block those situated in neurons. Moreover, LTCCs in the periphery are more abundant than in brain, and so it is inevitable that there will be side effects and risks related to LTCC blockade in the cardiovascular system. Indeed, effects on brain and behaviour may not be readily observable at tolerable doses of current LTCC antagonists.^84^ Hence, new LTCC antagonists, which can overcome the current limitations and are designed specifically for central nervous system indications are required.

A key advance in this regard would be the ability to target LTCC subtypes, which are enriched in the brain but which have low or minimal expression in the periphery. This is theoretically possible, given recent findings regarding the regulation, distribution and function, of LTCC subunit genes. There are no individual LTCC genes known, which are markedly more abundantly expressed in brain than elsewhere (although there are very few human data, and there are marked differences even between rats and mice).^96^ The existing data thus suggest that the answer to brain selectivity does not lie in gene-specific drugs; in any event, the fact that multiple LTCC subunits are implicated in bipolar disorder argues against this approach. Instead, improved selectivity may arise from the fact that individual LTCC genes are expressed as multiple variants (isoforms). For example, the human *CACNA1C* open reading frame is over 10 kb long, with at least 50 exons and over 40 predicted isoforms (arising from alternative splicing, transcriptional mechanisms and proteolytic processing). There is preliminary evidence in public databases, and in our own data (EMT, TAL and PJH, unpublished observations), that some of these isoforms show markedly higher expression in human brain than in human heart. As well as more clearly delineating these isoforms, it will be important to establish their properties, because it is already known that LTCC subunit isoforms vary in their functional characteristics.^97^ For example, alternative α_1_ subunit isoforms differ in their inactivation kinetics, voltage-dependence and—notably—their sensitivity to dihydropyridine-type LTCC antagonists.^84, 98^ A precedent for the clinical relevance of LTCC splice variants is provided by the *CACNA1B* (Ca_v_2.2) subunit of N-type calcium channels: a specific alternate exon is expressed in nociceptive neurons and is critical for pain signalling.^99, 100^ The β-subunit genes also encode multiple transcripts that show functional differences (for example, in their voltage-dependence and protein–protein interactions). In addition to these direct effects on LTCC function, specific isoforms of α_1_ and β LTCC subunit genes can regulate gene expression, with the C-termini of both Ca_V_1.2 and Ca_V_1.3 acting as transcription factors^101, 102^ and β LTCC subunits also being implicated in transcriptional regulation.^103^ These details highlight the potential for, but also the difficulties in, refining the molecular targets for a novel generation of brain-selective LTCC antagonists to be developed for use in bipolar disorder and other psychiatric conditions.

As and when new LTCC compounds are ready for testing in bipolar disorder, early evidence of target engagement (that is, effective blockade of brain LTCCs) will be valuable in order to inform about appropriate dosages, and to help de-risk their development. Such evidence can be acquired in experimental medicine studies of healthy volunteers. In this regard, magnetoencephalography may be of particular value. Magnetoencephalography signal reflects primarily the synchronous discharge of populations of cortical pyramidal neurons, and given the localisation of LTCCs on neuronal dendrites and their effects on dendritic depolarisation, it would be predicted that LTCC antagonism will produce a detectable effect on magnetoencephalography signal. Once target engagement has been shown, the next crucial step would be to confirm what effects, if any, novel LTCC antagonists have on mental, cognitive and circadian phenotypes, and the extent of cardiovascular and other ‘off-target' effects. Only once these initial studies have proved indication of efficacy and tolerability would trials in bipolar disorder or other psychiatric disorders be warranted.

In summary, despite over 30 years of use in bipolar disorder, LTCC antagonists have been tested neither carefully nor optimally in clinical trials, and hence it is still uncertain whether they have a role in the treatment of depressive or manic episodes, or in maintenance. As a result of recent genomic and cellular data, the therapeutic candidacy of this class of drugs in psychiatry has become considerably stronger. Large, carefully done and randomised studies are imperative in order to weigh the benefits and the risks of such treatments. We suggest that a renewed focus on LTCCs as targets, and the development of ‘brain-selective' LTCC ligands, could be one fruitful approach to innovative pharmacotherapy for bipolar disorder and related phenotypes.^104^

## Figures and Tables

**Table 1:** Characteristics of the six double-blind randomised trials included in the review

*Study ID*	*Verapamil (*N*) (daily dose)*	*Comparator B (*N*) (daily dose)*	*Setting and diagnosis*	*Usable data?*	*Cross over?*	*Follow up (weeks)*	*Previous long-term treatment*
Dubovsky^19^	Verapamil (7) (120 mg qds)	Placebo (7)	Manic inpatients (DSM III)	Y	Y	3½ (24 days)	Yes—six patients had a previous response to lithium
Garza-Trevino^20^	Verapamil (12) (80 mg qds)	Lithium (11) (0.75–1.0 mmol l^−1^)	Manic inpatients (DSM III-R)	Y	N	4	No information reported
Giannini^21^	Verapamil (10) (80 mg qds)	Lithium (10) (0.8–1.0 mmol l^−1^)	Manic patients (DSM III)	N	Y	26	Yes—all subjects with at least one past documented manic episode responsive to lithium carbonate within the previous 21 months. All patients stabilised with lithium within 3 weeks of episode and then maintained on lithium for a period ranging from 4–17 months
Janicak^22^	Verapamil (17) (160 mg tds)	Placebo (15)	Manic or mixed inpatients (DSM III-R)	Y	N	3	No clear information reported
Mallinger^23^	Verapamil (10) (160 mg tds)	Lithium (8) (0.8–1.2 mmol l^−1^)	Lithium-resistant manic in and outpatients (DSM-IV)	Y	N	3	Yes—all received open lithium for a minimum of 3 weeks and non-responders were randomly assigned to verapamil or continued lithium. Lithium was tapered down over 9 days (~25% per day) for those assigned to verapamil
Pal Singh^24^	Verapamil (25) (80 mg qds)	Lithium (25) (0.8–1.2 mmol l^−1^)	Manic inpatients (ICD 10)	Y	N	4	No information reported

Abbreviations: Y, yes; N, no.

**Table 2:** Rationale and considerations for the future development of LTCC antagonists for bipolar disorder (see text for details)

*Recent findings strengthening candidacy of LTCCs as therapeutic targets*	*Pharmacological and molecular considerations*
• *CACNA1C* locus shows genome-wide association to bipolar disorder, as well as to schizophrenia, major depression, working memory and sleep quality	• Blood-brain barrier penetration • Half-life
• Other LTCC subunit genes also show genome-wide association to bipolar disorder	• Evidence for target engagement in brain
• Rare variants in LTCC subunit genes are associated with bipolar disorder	• Selective targeting of brain-enriched isoforms to avoid cardiovascular side effects
• Neuron-like cells derived from bipolar disorder patients show altered calcium signalling	• Focus on relevant aspects of the bipolar phenotype, for example, mood stability, cognition, and sleep
• Neuron-like cells derived from subjects with *CACNA1C* risk genotype show increased gene expression and enhanced calcium signalling	• Long-term safety, for example, with regard to cancer risk

Abbreviations: LTCC, l-type calcium channel.
