# Supplementary material for: A systematic review of calcium channel antagonists in bipolar disorder and some considerations for their future development
Source: Mol Psychiatry. 2016 May 31;21(10):1324–32. doi: 10.1038/mp.2016.86 (PMC5030455; doi:10.1038/mp.2016.86)
Supplement: Supplementary Information [file mp201686x1.doc]

**A systematic review of calcium channel antagonists in bipolar disorder and some considerations for their future development**

***Supplementary information***

Andrea Cipriani, Kate Saunders, Mary-Jane Attenburrow, James Stefaniak, Priyanka Panchal, Sarah Stockton, Tracy A Lane,

Elizabeth M Tunbridge, John R Geddes, Paul J Harrison

**Appendix 1**

**Full search strategy**

| **Search**: ‘L-type’ calcium channel blockers for bipolar disorder | |
| --- | --- |
| **Interface**: OVID SP, Wiley, Ebsco host | **Databases**: Embase, Medline, PreMedline, PsycINFO, CDSR, DARE, CENTRAL, HTA, CINAHL |
| **Filters**: N/A | **Date Range (Embase, PsycINFO, CDSR, DARE, CENTRAL, HTA, CINAHL):** inception to 08.04.2015  **Date Range (PubMed, Medline, PreMedline):** inception to 15.02.2016 |
| **Hits**:  Undeduped: 2293  Deduped: 1665 | |

**Embase, Medline, PreMedline, PsycINFO – OVID SP interface**

| 1 | affective psychosis/ or exp bipolar disorder/ or mania/ or mood disorder/ |
| --- | --- |
| 2 | 1 use emez |
| 3 | affective disorders, psychotic/ or exp bipolar disorder/ or mood disorders/ |
| 4 | 3 use mesz |
| 5 | affective disorders/ or affective psychosis/ or exp bipolar disorder/ or exp mania/ |
| 6 | 5 use psyh |
| 7 | (((bipolar or bi polar) adj5 (disorder* or depress*)) or ((affective or mood) adj2 disorder*) or ((cyclothymi* or rapid or ultradian) adj5 cycl*) or hypomani* or mania* or manic* or mixed episode* or rcbd).tw. |
| 8 | or/2,4,6-7 |
| 9 | calcium channel.sh. |
| 10 | calcium channel blocking agent.sh. |
| 11 | calcium channel l type.sh. |
| 12 | or/9-11 use emez |
| 13 | calcium channel blockers.sh. |
| 14 | calcium channels.sh. |
| 15 | calcium channels, l-type.sh. |
| 16 | or/13-15 use mesz |
| 17 | channel blockers.sh. |
| 18 | calcium channel.sh. |
| 19 | or/17-18 use psyh |
| 20 | ((calcium adj2 (antagonist* or block* or channel* or inhibit*)) or ccb or ccbs or dhp receptor*).tw. |
| 21 | exp dihydropyridine derivative/ use emez |
| 22 | exp dihydropyridines/ use mesz |
| 23 | dihydropyridin*.tw. |
| 24 | phenylalkylamine.sh. use emez |
| 25 | phenylalkylamin*.tw. |
| 26 | benzothiazepine derivative.sh. use emez |
| 27 | benzothiazepin*.tw. |
| 28 | or/12,16,19-27 |
| 29 | amlodipin*.tw,sh. or (amlodipin* or amloc or amlopin* or amlor or astudal or istin* or levamlodipin* or norvasc).tw. |
| 30 | aranidipin*.tw,sh. or sapresta.tw. |
| 31 | azelnidipin*.tw,sh. or calblock.tw. |
| 32 | mepirodipin*.tw,sh. or (barnidipin* or cyress or hypoca or libradin* or vasexten).tw. |
| 33 | benidipin*.tw,sh. or (benidipinum or coniel).tw. |
| 34 | cilnidipin*.tw,sh. or (atelec or cilnidipin* or cinaldipin* or cinalong or cilacar or siscard).tw. |
| 35 | clevidipin*.tw,sh. or (clevelox or cleviprex).tw. |
| 36 | efonidipin*.tw,sh. or (finte or landel).tw. |
| 37 | felodipin*.tw,sh. or (agon or dilahex or dilofen or dilopin* or fedil* or felim or felo or felobal or felobeta or felocor or felodur or felogamma or felogard or felop or felopin* or felupuren or fensel or flodil* or hydac or keydipin* or lodistad or modip or munobal or nirmadil* or penedil* or perfudal or plendil* or preslow or prevex or renedil* or selepin* or splendil* or versant).tw. |
| 38 | isradipin*.tw,sh. or (dynacirc or icaz or carboxylate or isrodipin* or lomir or prescal or vascal).tw. |
| 39 | lacidipin*.tw,sh. or (caldin* or lacipil* or lacirex or motens).tw. |
| 40 | lercanidipin*.tw,sh. or (carmen or corifeo or lercadip or lercan or lerdip or masnidipin* or zanedip or zanidip).tw. |
| 41 | manidipin*.tw,sh. or (calslot or franidipin* or manyper).tw. |
| 42 | nicardipin*.tw,sh. or (antagonil* or barizin* or bionicard or cardene or cardepin* or cardibloc or cardipene or dacarel or dagan or flusemide or karden or lecibral or lincil* or loxen or lucenfal or nerdipin* or nicardal or nicardil* or nicarpin* or nicodel or nimicor or perdipin* or ranvil* or ridene or roxen or rycarden or rydene or saf card or vasodin* or vasonase).tw. |
| 43 | nifedipin*.tw,sh. or (adalat or adalate or adefin* or adipin* or afeditab or aldipin* or alonix or angibloc or angipec or antiblut or apo-nifed or aponifed or aprical or atanaal or calcheck or calcibloc or calcigard or calcilat or calgina or cardifen or cardilat or cardionorm or chronadalat or chronadalate or cipilat or citilat or coracten or cordafen or cordaflex or cordalat or cordicant or cordipen or cordipin* or corinfar or coronpin* or corotrend or denkifed or depin* or dignokonstant or dilafed or dipinkor or duranifin* or ecodipin* or emaberin* or fedcor or fedipin* or fenamon or fenigidin* or glopir or hadipin* or herlat or hexadilat or infedipin* or jutadilat or kemolat or korinfar or megalat or mifedipin* or moderat or myogard or nadipin* or nedipin* or nefedipin* or nelapin* or nifangin* or nifar or nifdemin* or nifebene or nifecard or nifecor or nifedepat or nifedicor or nifedilat or nifedin* or nifedin* or nifedipat or nifedipres or nifedirex or nifehexal or nifelat or nifensar or nifepidin* or nifestad or nifical or nificard or nifidin* or nifipen or nipin* or normadil* or novo nifedin* or novonifedin* or nyefax or nypin* or odipin* or orix or osmo-adalat or phenygidin* or pidilat or procardia or ronian or sepamit or slofedipin* or tibricol or unidipin* or vascard or vasdalat or zenusin*).tw. |
| 44 | nilvadipin*.tw,sh. or (escor or nilvadil* or nivadil* or nivadipin*).tw. |
| 45 | nimodipin*.tw,sh. or (admon or brainal or calnit or eugerial or grifonimod or kenesil* or kenzolol or modus or nidip or nimodilat or nimotop or nisom or nymalize or periplum or remontal or tropocer or vasoflex or vasotop).tw. |
| 46 | nisoldipin*.tw,sh. or (angiolat or baymycard or corasol or nisoldin* or sular or syscor).tw. |
| 47 | nitrendipin*.tw,sh. or (balminil* or baylotensin* or bayotensin* or baypresol or baypress or gericin* or jutapress or nidrel or niprin* or nitre abz or nitren acis or nitrendepat or nitrendidoc or nitrensal or nitrepress or nitrepuren or trendinol or vastensium).tw. |
| 48 | pranidipin*.tw,sh. or acalas.tw. |
| 49 | riodipin*.tw,sh. or (foridon* or phoridon* or riosidin* or ryodipin* or ryosidin*).tw. |
| 50 | trimetazidin*.tw,sh. or (centrophen* or idaptan or preductal or trimethazidin* or trimethazidin* or vastarel).tw. |
| 51 | or/29-50 |
| 52 | anipamil*.tw,sh. |
| 53 | devapamil*.tw,sh. or (demethoxyverapamil* or desmethoxyverapamil*).tw. |
| 54 | falipamil*.tw,sh. |
| 55 | gallopamil*.tw,sh. or (compound d 600 or compound d600 or galcan or gallopamil* or methoxyverapamil* or procorum or verapamil*).tw. |
| 56 | tiapamil*.hw,tw. or (dimeditiapramin* or larocord or thiapamil* or tiapamil* or verocainide or verocainin*).tw. |
| 57 | verapamil*.tw,sh. or (apo-verap or apoacor or arpamyl or azupamil* or berkatens or bosoptin* or calan or calaptin* or cardiagutt or cardibeltin* or cardiolen or cardiover or caveril* or cintsu or civicor or coer 24 or coraver or cordilat or cordilox or corpamil* or covera or dexverapamil* or dignover or dilacoran or dilacoron or durasoptin* or falicard or finoptin* or flamon or geangin* or hexasoptin* or ikacor or ikakor or ikapress or iproveratril* or iso-card or isoptin* or izoptin* or manidon or napamil* or novapamyl or novo-veramil or novopressan or phynoptin* or quasar or ravamil* or securon or univer or vasolan or vasomil* or vasopten or verabeta or veracaps or veracor or verahexal or veraloc or veramex or veramil *or verapamil* or verapin* or verapress or veratad or verdilac or verelan or verexamil* or veroptin* or verapamil* or vetrimil * or vortac or zolvera).tw. |
| 58 | or/52-57 |
| 59 | clentiazem.tw,sh. or (clentiazem or logna).tw. |
| 60 | diltiazem.tw,sh. or (acalix or adizem or aldizem or altiazem or anginyl or angiotrofen or angiotrofin* or angiozem or angizem or angoral or anoheal or anzem or apo-diltiazem or auscard or balcor or beatizem or bi-tildiem or blocalcin* or britiazim or bruzem or calcicard or calnurs or cardcal or cardiazem or cardiben or cardiem or cardil* or cardiosta or cardium or cardizem or carex or cartia or cascor or cirilen or cirilen ap or cis diltiazem or coras or cordizem or dazil or deltazen or diacor or diatal or dilacor or diladel or dilatam or dilatame or dilcard or dilcardia or dilem or dilfar or dilgard or diloc or dilren or dilren*1 or dilso or dilt-cd or diltahexal or diltam or diltan or diltelan or diltia or diltiamax or diltiasyn or diltiazem or diltime or diltzac or dilzanton or dilzem or dilzene or dilzereal 90 retard or dilzicardin* or dinisor or dodexen or dyalac or entrydil or filazem or gadoserin* or grifodilzem or hagen or helsibon or herben or herbesser or herbessor or hesor or incoril or kaizem or lacerol or levodex or levozem or lytelsen or masdil* or miocardie or mono-tildiem or monotildiem or myonil or pazeadin* or presoken or surazem or tazem or taztia or tiadil* or tiamate or tiazac or tilazem or tildiem or trans diltiazem or vasmulax or vasocardol or wentizem or zandil* or zemtrial zildem or ziruvate).tw. |
| 61 | or/59-60 |
| 62 | or/28,51,58,61 |
| 63 | 8 and 62 |

**Cochrane Library: CDSR, DARE, HTA, CENTRAL – Wiley interface**

#1 mesh descriptor: [bipolar disorder] explode all trees

#2 mesh descriptor: [mood disorders] this term only

#3 mesh descriptor: [affective disorders, psychotic] this term only

#4 ((("bi polar " or bipolar) near/5 (disorder* or depress*)) or ((affective or mood) near/2 disorder*) or ((cyclothymi* or rapid or ultradian) near/1 cycl*) or hypomani* or mania* or manic* or "mixed episode*" or rcbd)

#5 #1 or #2 or #3 or #4

#6 mesh descriptor: [calcium channel blockers] this term only

#7 mesh descriptor: [calcium channels, l-type] this term only

#8 mesh descriptor: [calcium channels] this term only

#9 ((calcium near/2 (antagonist* or block* or inhibit*)) or ccb or ccbs):ti,ab,kw

#10 mesh descriptor: [dihydropyridines] explode all trees

#11 dihydropyridine*

#12 phenylalkylamine*

#13 benzothiazepin*

#14 #6 or #7 or #8 or #9 or #10 or #11 or #12 or #13

#15 (amlodipin* or amloc or amlopin* or amlor or astudal or istin* or levamlodipin* or norvasc):ti,ab,kw

#16 (aranidipin* or sapresta):ti,ab,kw

#17 (azelnidipin* or calblock):ti,ab,kw

#18 (mepirodipin* or barnidipin* or cyress or hypoca or libradin* or vasexten):ti,ab,kw

#19 (benidipin* or benidipinum or coniel):ti,ab,kw

#20 (cilnidipin* or atelec or cilnidipin* or cinaldipin* or cinalong or cilacar or siscard):ti,ab,kw

#21 (clevidipin* or clevelox or cleviprex):ti,ab,kw

#22 (efonidipin* or finte or landel):ti,ab,kw

#23 (felodipin* or agon or dilahex or dilofen or dilopin* or fedil* or felim or felo or felobal or felobeta or felocor or felodur or felogamma or felogard or felop or felopin* or felupuren or fensel or flodil* or hydac or keydipin* or lodistad or modip or munobal or nirmadil* or penedil* or perfudal or plendil* or preslow or prevex or renedil* or selepin* or splendil* or versant):ti,ab,kw

#24 (isradipin* or dynacirc or icaz or carboxylate or isrodipin* or lomir or prescal or vascal):ti,ab,kw

#25 (lacidipin* or caldin* or lacipil* or lacirex or motens):ti,ab,kw

#26 (lercanidipin* or carmen or corifeo or lercadip or lercan or lerdip or masnidipin* or zanedip or zanidip):ti,ab,kw

#27 (manidipin* or calslot or franidipin* or manyper):ti,ab,kw

#28 (nicardipin* or antagonil* or barizin* or bionicard or cardene or cardepin* or cardibloc or cardipene or dacarel or dagan or flusemid* or karden or lecibral or lincil or loxen or lucenfal or nerdipin* or nicardal or nicardil* or nicarpin* or nicodel or nimicor or perdipin* or ranvil or ridene or roxen or rycarden or rydene or "saf card" or vasodin* or vasonase):ti,ab,kw

#29 (nifedipin* or adalat or adalate or adefin* or adipin* or afeditab or aldipin* or alonix or angibloc or angipec or antiblut or "apo-nifed" or aponifed or aprical or atanaal or calcheck or calcibloc or calcigard or calcilat or calgina or cardifen or cardilat or cardionorm or chronadalat or chronadalate or cipilat or citilat or coracten or cordafen or cordaflex or cordalat or cordicant or cordipen or cordipin* or corinfar or coronpin* or corotrend or denkifed or depin or dignokonstant or dilafed or dipinkor or duranifin* or ecodipin* or emaberin* or fedcor or fedipin* or fenamon or fenigidin* or glopir or hadipin* or herlat or hexadilat or infedipin* or jutadilat or kemolat or korinfar or megalat or mifedipin* or moderat or myogard or nadipin* or nedipin* or nefedipin* or nelapin* or nifangin* or nifar or nifdemin* or nifeben* or nifecard or nifecor or nifedepat or nifedicor or nifedilat or nifedin* or nifedipat or nifedipres or nifedirex or nifehexal or nifelat or nifensar or nifepidin* or nifestad or nifical or nificard or nifidin* or nifipen or nipin or normadil* or novonifedin* or nyefax or nypin* or odipin* or orix or "osmo-adalat" or phenygidin* or pidilat or procardia or ronian or sepamit or slofedipine or tibricol or unidipine or vascard or vasdalat or zenusin*):ti,ab,kw

#30 (nilvadipin* or escor or nilvadil* or nivadil* or nivadipin*):ti,ab,kw

#31 (nimodipin* or admon or brainal or calnit or eugerial or grifonimod or kenesil* or kenzolol or modus or nidip or nimodilat or nimotop or nisom or nymalize or periplum or remontal or tropocer or vasoflex or vasotop):ti,ab,kw

#32 (nisoldipin* or angiolat or baymycard or corasol or nisoldin* or sular or syscor):ti,ab,kw

#33 (nitrendipin* or balminil* or baylotensin* or bayotensin* or baypresol or baypress or gericin or jutapress or nidrel or niprina or "nitre abz" or "nitren acis" or nitrendepat or nitrendidoc or nitrensal or nitrepress or nitrepuren or trendinol or vastensium):ti,ab,kw

#34 (pranidipin* or acalas):ti,ab,kw

#35 (riodipin* or foridon* or phoridon* or riosidin* or ryodipin* or ryosidin*):ti,ab,kw

#36 (trimetazidin* or centrophene or idaptan or preductal or trimethazidin* or vastarel):ti,ab,kw

#37 #15 or #16 or #17 or #18 or #19 or #20 or #21 or #22 or #23 or #24 or #25 or #26 or #27 or #28 or #29 or #30 or #31 or #32 or #33 or #34 or #35 or #36

#38 anipamil*:ti,ab,kw

#39 (devapamil* or demethoxyverapamil* or desmethoxyverapamil*):ti,ab,kw

#40 falipamil*:ti,ab,kw

#41 (gallopamil or "compound d 600" or "compound d600" or galcan or gallopamil* or methoxyverapamil* or procorum or verapamil*):ti,ab,kw

#42 (tiapamil* or dimeditiapramin* or larocord or thiapamil* or tiapamil* or verocainide or verocainin*):ti,ab,kw

#43 (verapamil* or "apo-verap" or apoacor or arpamyl or azupamil* or berkatens or bosoptin* or calan or calaptin* or cardiagutt or cardibeltin* or cardiolen or cardiover or caveril* or cintsu or civicor or "coer 24" or coraver or cordilat or cordilox or corpamil* or covera or dexverapamil* or dignover or dilacoran or dilacoron or durasoptin* or falicard or finoptin* or flamon or geangin* or hexasoptin* or ikacor or ikakor or ikapress or iproveratril* or "iso-card" or isoptin* or izoptin* or manidon or napamil* or novapamyl or "novo-veramil*" or novopressan or phynoptin* or quasar or ravamil* or securon or univer or vasolan or vasomil* or vasopten or verabeta or veracaps or veracor or verahexal or veraloc or veramex or veramil* or verapamil* or verapin* or verapress or veratad or verdilac or verelan or verexamil* or veroptin* or verapamil* or vetrimil* or vortac or zolvera):ti,ab,kw

#44 #38 or #39 or #40 or #41 or #42 or #43

#45 (clentiazem* or logna):ti,ab,kw

#46 (diltiazem* or acalix or adizem or aldizem or altiazem or anginyl or angiotrofen or angiotrofin* or angiozem or angizem or angoral or anoheal or anzem or auscard or balcor or beatizem or "bi-tildiem" or blocalcin* or britiazim or bruzem or calcicard or calnurs or cardcal or cardiazem or cardiben or cardiem or cardil* or cardiosta or cardium or cardizem or carex or cartia or cascor or cirilen or "cis diltiazem" or coras or cordizem or dazil* or deltazen or diacor or diatal or dilacor or diladel or dilatam or dilatame or dilcard or dilcardia or dilem or dilfar or dilgard or diloc or dilren or dilren* or dilso or "dilt-cd" or diltahexal or diltam or diltan or diltelan or diltia or diltiamax or diltiasyn or diltiazem or diltime or diltzac or dilzanton or dilzem or dilzene or dilzereal r dilzicardin* or dinisor or dodexen or dyalac or entrydil or filazem or gadoserin* or grifodilzem or hagen or helsibon or herben or herbesser or herbessor or hesor or incoril or kaizem or lacerol or levodex or levozem or lytelsen or masdil* or miocardie or "mono-tildiem" or monotildiem or myonil* or pazeadin* or presoken or surazem or tazem or taztia or tiadil* or tiamate or tiazac or tilazem or tildiem or vasmulax or vasocardol or wentizem or zandil* or zemtrial or zildem or ziruvate):ti,ab,kw

#47 #45 or #46

#48 #14 or #37 or #44 or #47

#49 #5 and #48

**CINAHL – Ebsco Host**

Top of Form

| **#** | **query** |
| --- | --- |
| s28 | s4 and s27 |
| s27 | s11 or s20 or s23 or s26 |
| s26 | s24 or s25 |
| s25 | tx ( (clentiazem* or logna) ) and tx ( (diltiazem* or acalix or adizem or aldizem or altiazem or anginyl or angiotrofen or angiotrofin* or angiozem or angizem or angoral or anoheal or anzem or auscard or balcor or beatizem or "bi-tildiem" or blocalcin* or britiazim or bruzem or calcicard or calnurs or cardcal or cardiazem or cardiben or cardiem or cardil* or cardiosta or cardium or cardizem or carex or cartia or cascor or cirilen or "cis diltiazem" or coras or cordizem or dazil* or deltazen or diacor or diatal or dilacor or diladel or dilatam or dilatame or dilcard or dilcardia or dilem or dilfar or dilgard or diloc or dilren or dilren* or dilso or "dilt-cd" or diltahexal or diltam or diltan or diltelan or diltia or diltiamax or diltiasyn or diltiazem or diltime or diltzac or dilzanton or dilzem or dilzene or dilzereal r dilzicardin* or dinisor or dodexen or dyalac or entrydil or filazem or gadoserin* or grifodilzem or hagen or helsibon or herben or herbesser or herbessor or hesor or incoril or kaizem or lacerol or levodex or levozem or lytelsen or masdil* or miocardie or "mono-tildiem" or monotildiem or myonil* or pazeadin* or presoken or surazem or tazem or taztia or tiadil* or tiamate or tiazac or tilazem or tildiem or vasmulax or vasocardol or wentizem or zandil* or zemtrial or zildem or ziruvate) ) |
| s24 | tx ( (clentiazem* or logna) ) and tx ( (diltiazem* or acalix or adizem or aldizem or altiazem or anginyl or angiotrofen or angiotrofin* or angiozem or angizem or angoral or anoheal or anzem or auscard or balcor or beatizem or "bi-tildiem" or blocalcin* or britiazim or bruzem or calcicard or calnurs or cardcal or cardiazem or cardiben or cardiem or cardil* or cardiosta or cardium or cardizem or carex or cartia or cascor or cirilen or "cis diltiazem" or coras or cordizem or dazil* or deltazen or diacor or diatal or dilacor or diladel or dilatam or dilatame or dilcard or dilcardia or dilem or dilfar or dilgard or diloc or dilren or dilren* or dilso or "dilt-cd" or diltahexal or diltam or diltan or diltelan or diltia or diltiamax or diltiasyn or diltiazem or diltime or diltzac or dilzanton or dilzem or dilzene or dilzereal r dilzicardin* or dinisor or dodexen or dyalac or entrydil or filazem or gadoserin* or grifodilzem or hagen or helsibon or herben or herbesser or herbessor or hesor or incoril or kaizem or lacerol or levodex or levozem or lytelsen or masdil* or miocardie or "mono-tildiem" or monotildiem or myonil* or pazeadin* or presoken or surazem or tazem or taztia or tiadil* or tiamate or tiazac or tilazem or tildiem or vasmulax or vasocardol or wentizem or zandil* or zemtrial or zildem or ziruvate) ) |
| s23 | s21 or s22 |
| s22 | tx ( (gallopamil or "compound d 600" or "compound d600" or galcan or gallopamil* or methoxyverapamil* or procorum or verapamil*) ) or tx ( (tiapamil* or dimeditiapramin* or larocord or thiapamil* or tiapamil* or verocainide or verocainin*) ) or tx ( (verapamil* or "apo-verap" or apoacor or arpamyl or azupamil* or berkatens or bosoptin* or calan or calaptin* or cardiagutt or cardibeltin* or cardiolen or cardiover or caveril* or cintsu or civicor or "coer 24" or coraver or cordilat or cordilox or corpamil* or covera or dexverapamil* or dignover or dilacoran or dilacoron or durasoptin* or falicard or finoptin* or flamon or geangin* or hexasoptin* or ikacor or ikakor or ikapress or iproveratril* or "iso-card" or isoptin* or izoptin* or manidon or napamil* or novapamyl or "novo-veramil*" or novopressan or phynoptin* or quasar or ravamil* or securon or univer or vasolan or vasomil* or vasopten or verabeta or veracaps or veracor or verahexal or veraloc or veramex or veramil* or verapamil* or verapin* or verapress or veratad or verdilac or verelan or verexamil* or veroptin* or verapamil* or vetrimil* or vortac or zolvera) ) |
| s21 | tx anipamil* or tx ( (devapamil* or demethoxyverapamil* or desmethoxyverapamil*) ) or tx falipamil* |
| s20 | s12 or s13 or s14 or s15 or s16 or s17 or s18 or s19 |
| s19 | (trimetazidin* or centrophene or idaptan or preductal or trimethazidin* or vastarel) |
| s18 | ( (nitrendipin* or balminil* or baylotensin* or bayotensin* or baypresol or baypress or gericin or jutapress or nidrel or niprina or "nitre abz" or "nitren acis" or nitrendepat or nitrendidoc or nitrensal or nitrepress or nitrepuren or trendinol or vastensium) ) or ( (pranidipin* or acalas) ) or ( (riodipin* or foridon* or phoridon* or riosidin* or ryodipin* or ryosidin*) ) |
| s17 | ( (nilvadipin* or escor or nilvadil* or nivadil* or nivadipin*) ) or ( (nimodipin* or admon or brainal or calnit or eugerial or grifonimod or kenesil* or kenzolol or modus or nidip or nimodilat or nimotop or nisom or nymalize or periplum or remontal or tropocer or vasoflex or vasotop) ) or ( (nisoldipin* or angiolat or baymycard or corasol or nisoldin* or sular or syscor) ) |
| s16 | ( (manidipin* or calslot or franidipin* or manyper) ) or ( (nicardipin* or antagonil* or barizin* or bionicard or cardene or cardepin* or cardibloc or cardipene or dacarel or dagan or flusemid* or karden or lecibral or lincil or loxen or lucenfal or nerdipin* or nicardal or nicardil* or nicarpin* or nicodel or nimicor or perdipin* or ranvil or ridene or roxen or rycarden or rydene or "saf card" or vasodin* or vasonase) ) or ( (nifedipin* or adalat or adalate or adefin* or adipin* or afeditab or aldipin* or alonix or angibloc or angipec or antiblut or "apo-nifed" or aponifed or aprical or atanaal or calcheck or calcibloc or calcigard or calcilat or calgina or cardifen or cardilat or cardionorm or chronadalat or chronadalate or cipilat or citilat or coracten or cordafen or cordaflex or cordalat or cordicant or cordipen or cordipin* or corinfar or coronpin* or corotrend or denkifed or depin or dignokonstant or dilafed or dipinkor or duranifin* or ecodipin* or emaberin* or fedcor or fedipin* or fenamon or fenigidin* or glopir or hadipin* or herlat or hexadilat or infedipin* or jutadilat or kemolat or korinfar or megalat or mifedipin* or moderat or myogard or nadipin* or nedipin* or nefedipin* or nelapin* or nifangin* or nifar or nifdemin* or nifeben* or nifecard or nifecor or nifedepat or nifedicor or nifedilat or nifedin* or nifedipat or nifedipres or nifedirex or nifehexal or nifelat or nifensar or nifepidin* or nifestad or nifical or nificard or nifidin* or nifipen or nipin or normadil* or novonifedin* or nyefax or nypin* or odipin* or orix or "osmo-adalat" or phenygidin* or pidilat or procardia or ronian or sepamit or slofedipine or tibricol or unidipine or vascard or vasdalat or zenusin*) ) |
| s15 | ( (isradipin* or dynacirc or icaz or carboxylate or isrodipin* or lomir or prescal or vascal) ) or ( (lacidipin* or caldin* or lacipil* or lacirex or motens) ) or ( (lercanidipin* or carmen or corifeo or lercadip or lercan or lerdip or masnidipin* or zanedip or zanidip) ) |
| s14 | ( (clevidipin* or clevelox or cleviprex) ) or ( (efonidipin* or finte or landel) ) or ( (felodipin* or agon or dilahex or dilofen or dilopin* or fedil* or felim or felo or felobal or felobeta or felocor or felodur or felogamma or felogard or felop or felopin* or felupuren or fensel or flodil* or hydac or keydipin* or lodistad or modip or munobal or nirmadil* or penedil* or perfudal or plendil* or preslow or prevex or renedil* or selepin* or splendil* or versant) ) |
| s13 | ( (mepirodipin* or barnidipin* or cyress or hypoca or libradin* or vasexten) ) or ( (benidipin* or benidipinum or coniel) ) or ( (cilnidipin* or atelec or cilnidipin* or cinaldipin* or cinalong or cilacar or siscard) ) |
| s12 | ( (amlodipin* or amloc or amlopin* or amlor or astudal or istin* or levamlodipin* or norvasc) ) or ( (aranidipin* or sapresta) ) or ( (azelnidipin* or calblock) ) |
| s11 | s5 or s6 or s7 or s8 or s9 or s10 |
| s10 | tx benzothiazepin* |
| s9 | tx phenylalkylamine* |
| s8 | tx dihydropyridine* |
| s7 | ((calcium n2 (antagonist* or block* or channel* or inhibit*)) or ccb or ccbs or "dhp receptor*") |
| s6 | (mh "calcium channel agonists") |
| s5 | (mh "calcium channel blockers") |
| s4 | s1 or s2 or s3 |
| s3 | tx ((("bi polar " or bipolar) n5 (disorder* or depress*)) or ((affective or mood) n2 disorder*) or ((cyclothymi* or rapid or ultradian) n1 cycl*) or hypomani* or mania* or manic* or "mixed episode*" or rcbd) |
| s2 | (mh "affective disorders") |
| s1 | (mh "affective disorders, psychotic+") |

Bottom of Form

**Appendix 2**

**Risk of bias tool**

We followed the recommended approach for assessing risk of bias in studies included in Cochrane reviews. It is a two-part tool, addressing the six specific domains (namely sequence generation, allocation concealment, blinding, incomplete outcome data, selective outcome reporting and ‘other issues’). Two of the items (adequacy of sequence generation and allocation concealment) assess the strength of the randomization process in preventing selection bias in the assignment of participants to interventions; the third item (blinding) assesses the influence of performance bias on the study results and the fourth the likelihood of incomplete outcome data, which raise the possibility of bias in effect estimates. The fifth item assesses selective reporting, the tendency to preferentially report statistically significant outcomes (this item requires a comparison of published data with trial protocols, when such are available). The final item refers to other sources of bias that are relevant in certain circumstances, such as, for example, sponsorship bias.

Each domain includes one or more specific entries in a ‘Risk of bias’ table. Within each entry, the first part of the tool involves describing what was reported to have happened in the study. The second part of the tool involves assigning a judgement relating to the risk of bias for that entry. This is achieved by answering a pre-specified question about the adequacy of the study in relation to the entry, such that a judgement of ‘Yes’ indicates low risk of bias, ‘No’ indicates high risk of bias, and ‘Unclear’ indicates unclear or unknown risk of bias.

| **Domain** | **Description** | **Review authors’ judgement** |
| --- | --- | --- |
| **Sequence generation.** | Describe the method used to generate the allocation sequence in sufficient detail to allow an assessment of whether it should produce comparable groups. | *Was the allocation sequence adequately generated?* |
| **Allocation concealment.** | Describe the method used to conceal the allocation sequence in sufficient detail to determine whether intervention allocations could have been foreseen in advance of, or during, enrolment. | *Was allocation adequately concealed?* |
| **Blinding of participants, personnel and outcome assessors** | Describe all measures used, if any, to blind study participants and personnel from knowledge of which intervention a participant received. Provide any information relating to whether the intended blinding was effective. | *Was knowledge of the allocated intervention adequately prevented during the study?* |
| **Incomplete outcome data** *Assessments should be made for each main outcome (or class of outcomes).* | Describe the completeness of outcome data for each main outcome, including attrition and exclusions from the analysis. State whether attrition and exclusions were reported, the numbers in each intervention group (compared with total randomized participants), reasons for attrition/exclusions where reported, and any re-inclusions in analyses performed by the review authors. | *Were incomplete outcome data adequately addressed?* |
| **Selective outcome reporting.** | State how the possibility of selective outcome reporting was examined by the review authors, and what was found. | *Are reports of the study free of suggestion of selective outcome reporting?* |
| **Other sources of bias.** | State any important concerns about bias not addressed in the other domains in the tool. | *Was the study apparently free of other problems that could put it at a high risk of bias?* |

**Risk of bias summary: it is a summary table of review authors' judgments for each risk of bias item for each study.**


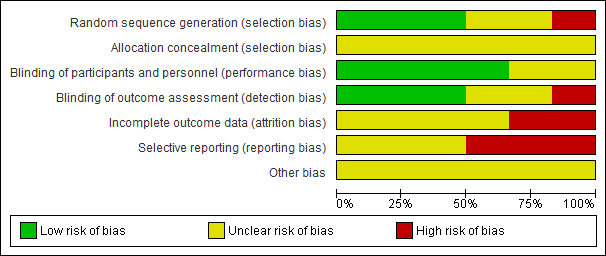


**Appendix 3**

**Characteristics of studies reporting adverse events**

| **Study (Author, Year)** | **Class** | **Agent & final dose** | **N** | **Methodology** | **Diagnosis & mood state** | **Outcome measures (where stated)** | **Follow-up period** | **Findings** | **Adverse Events** |
| --- | --- | --- | --- | --- | --- | --- | --- | --- | --- |
| **NON RANDOMISED DOUBLE BLIND TRIALS** | | | | | | | | | |
| Aldenhoff 1986 | P | D600  75mg | 10 | Double blind placebo controlled trial. Non randomised | DSM III Mania  Hospitalised cohort | GAS  BRMS  IMPS | 15 days | Statistically significant improvement in IMPS motor disturbance subscale | One participant required additional psychotropic medication for uncontrolled aggressiveness |
| Dose 1986 | P | Verapamil  320-480mg | 8 | Double blind A-B-A placebo controlled study | Manic or schizomanic syndrome | IMPS | 7 days | Average reduction in IMPS of 30% | “Slight changes in blood pressure, heart rates and digestion” |
| **OBSERVATIONAL STUDIES** | | | | | | | | | |
| Barton 1987 | P | Verapamil  Acute mania 80mg tds  Recurrent episodes 240-320mg  Pharmacological mania 80mg tds – 80mg qds | 14 | Open label study  Adjunctive treatment  Community sample | RDC bipolar-I (N=10), bipolar-II (N=3), schizoaff. disorder (N=1)  Mania (N=8)  Recurrent bipolar episodes (N=4)  Pharmacological mania (N=2) | YMRS | Acute mania: 21 days  Maintenance: 18 months  Pharmacological mania: 14 days | No improvement in acute mania  2 had mild improvement in recurrence of episodes  In those with pharmacological mania Verapamil prevented antidepressant induced mania | Dysphoric feelings  Headache  Constipation |
| Brunet 1990 | D | Nimodipine  360mg 4 hourly | 6 | Open label study | DSM III R bipolar disorder: acute mania, hospitalised | BRMS  BPRS | 7 days | Significant improvements in Bech scale (p<0.001) and BPRS (p<0.02) | Sleep disturbance (may be due to might time administration of the drug) |
| Caillard 1985 | B | Diltiazem  120mg-360mg | 5 | Open label study | DSM III Mania  Hospitalised | MSRS | 14 days | Significant improvements in MSRS >70% in all patients | Headache (N=1)  Vertigo without orthostatic hypotension (N=1)  Transient oedema of extremities (N=1) |
| Lenzi 1995 | P | Verapamil  400mg | 15 | Open label study  Adjunctive chlorpromazine permitted | DSM IIIR mania  Hospitalised cohort | BPRS  CGI | 21 days | 1 patient responded to verapamil monotherapy.  2 required night time chlorpromazine. 12 required higher doses of chlorpromazine  Improvements in BPRS by day 10. | Sweating (N=8)  Dizziness (N=2)  Tachycardia (N=5)  Constipation (N=5)  Blurred vision (N=5)  Hypotension (N=1) |
| Ostacher 2014 | D | Isradipine  5mg bd | 12 | Open label study  Adjunctive treatment | DSM IV bipolar I disorder (N=  DSM IV bipolar II disorder (N=  Acute depression | MADRS  QUIDS-SR  CGI-S  YMRS | 8 weeks | Mean MADRS decrease of 2.1 /wk. Mean QUIDS-SR decrease of 1.0/week. Mean CGI-S decrease of 0.18/wk. No significant change in YMRS noted. | Initial decrease in diastolic blood pressure and increased heart rate. Both returned to normal at 4 weeks. |
| Silverstone 2000 | B | Diltiazem  60mg – 120mg bd. Adjunctive treatment | 8 | Retrospective analysis of clinical outcomes | DSM IV bipolar disorder  Community sample | DSMIV diagnostic criteria | 6 months | Significant treatment between pre and post treatment symptom scores (F=88.2. p<0.0001) | Nausea (N=2)  Headache (N=1) |
| Snedkova  1997 | B | Nifedipine | 26 | Open label study  Lithium comparator | Affective and schizoaffective psychoses | Number of mood episodes.  Number of hospital admissions | 2 years | 46.8% reduction in duration of mood episodes  31.7% reduction in frequency of mood episodes  52.2% reduction in hospital admissions. | Dry mouth (N=4)  Peripheral vasodilation (N=3)  Iridocyclitis (N=1)  Headaches (N=4)  Digestive problems (N=4)  Sleep disturbance (N=4)  Leg oedema (N=1) |
| **CASE REPORTS** | | | | | | | | | |
| Deicken 1990 | P | Verapamil 320mg in divided doses Adjunctive treatment | 1 | Case report  44 yr old male  Treatment resistant | DSM IIIR bipolar disorder  Acute depression | BDI  HAMD | 9 months | No further depressive episodes during the follow-up period. | Intermittent mild headaches |
| Dubovsky 1987 | P | Verapamil  P1. 480mg  P2. 320mg | 2 | Case reports  Adjunctive treatment | Bipolar disorder  P1. Rapid cycling  P2. Acute mania |  | 21 days | P1. No improvement  P2. Mania improved | P1. Sinus bradycardia  P2. Sinus bradycardia with class 1 atrioventricular ectopy. Died of MI 3 days later |
| Gitlin 1984 | P | Verapamil  80mg tds | 1 | Case report  32 yr old woman  Adjunctive treatment | Bipolar disorder  Treatment resistant | BDI  BPRS | 15months | Euthymic except 4 brief periods of hypomania from commencing verapamil | Headaches  Constipation |
| Helmuth 1989 | P | Verapamil  120mg | 1 | Case report  Adjunctive treatment | DSM IIIR mania |  | 4 days | Manic symptoms resolved following addition of verapamil. Symptoms re-emerged on withdrawal | Involuntary choreoathetoid movements. Resolved on withdrawal of verapamil. |
| Jacobsen 1987 | P | Verapamil  80mg qds reduced to bd on reintroduction. | 1 | Case report  24 yr old female | Rapid cycling bipolar disorder |  | 7 days | No improvement in mood cycling until the introduction of amitriptyline 25mg and decrease in verapamil to 160mg. | Toxic delirium at 7 days. Symptoms re-emerged following discontinuation and reinstitution of verapamil |
| Price 1986 | P | Verapamil  80mg tds | 1 | Case report  54 yr old woman  Adjunct to carbamazepine | Manic depressive illness |  | 7 days | None reported | Persistent nausea, ataxia and incoordination. Mild memory impairment. All resolved on withdrawal of verapamil |
| Price 1988 | P | Verapamil  80mg tds | 1 | Case report  42 yr old woman  Adjunct to lithium | Rapid cycling bipolar disorder |  | 9 days | At 7 days no longer exhibited pressured speech, insomnia or grandiosity | Nausea, vomiting, muscular weakness, ataxia, and “ringing of the ears.” Ataxic gait. Complete resolution following withdrawal of verapamil. Re-challenge with verapamil provoked re-emergence of side effects. |
| Wisner 2002 | P | Verapamil  480mg maximum | 37 | Case series  Monotherapy | DSM IV bipolar disorder: mania or hypomania (N=2); mixed affective state (N=9); acute depression (N=18); euthymic (N=9); pregnant women (N=29) | HAMD  MRS BRMS  IDD | Minimum 1 month | 2/2 with mania responded  7/9 with mixed states responded  7/18 with depression responded  6/9 euthymic women remained well | Headache (N=1)  Rash (N=1)  Palpitations (N=1) |

**Legend.** Abbreviations: P= Phenilalkylamines, B= Benzothiazepines, D= Dihydropyridines, BDI – Beck Depression inventory, BPRS – Brief psychiatric rating scale, BMMRS – Beigel and Murphy mania rating scale. BRMS - Bech-rafaelsen mania rating scale, CGI – Clinical Global Index of severity, GAF – Global assessment of function, GAS - Global assessment scale, HAMD - Hamilton depression rating scale , IDD – Inventory to diagnose depression, IMPS – Inpatient-multidimensional psychiatric scale, MADRS - Montgomery-Asberg depression rating scale, MRS – Mania rating scale, MSRS - Manic state rating scale, QUIDS-SR - Quick inventory of depression score self-report, YMRS – Young mania rating scale.

**Appendix 4**

**Characteristics of all studies identified where calcium channel antagonists have been used in bipolar disorder**

| **Study (Author, Year)** | **Class** | **Agent & final dose** | **N** | | **Methodology** | | **Diagnosis & mood state** | | **Outcome measures (where stated)** | | **Follow-up period** | | **Findings** | | **Adverse Events** | |
| --- | --- | --- | --- | --- | --- | --- | --- | --- | --- | --- | --- | --- | --- | --- | --- | --- |
| **Randomised double blind trials** | | | | | | | | | | | | | | | |  |
| Dubovsky 1986[1] | P | Verapamil 120mg qds | 7 | | Double blind randomised placebo control trial  Crossover design | | DSM III bipolar disorder: acute mania  Hospitalised cohort | | MSRS  BPRS | | 24 days | | Significant reduction in MSRS compared with placebo | | Not reported | |
| Garza-Trevino 1992[2] | P | Verapamil  D1: 80mg bd,  D2-3: 80mg tds  D4-study end: 80mg qds | 20 | | Double blind randomised control trial  Lithium comparator | | DSM III mania  Hospitalised cohort | | Petterson Mania Scale  BPRS  CGI | | 28 days | | No difference between groups on Petterson Mania Scale or CGI | | Constipation (N=1) | |
| Giannini 1987[3] | P | Verapamil  80mg qds | 10 | | Double blind randomised control trial  Lithium comparator  Crossover design | | DSM III bipolar disorder: acute mania  Hospitalised cohort | | BPRS | | 180 days | | No difference between groups | | Not reported | |
| Janicak 1998[4] | P | Verapamil  D1: 160mg  D2: 240 mg  D3-6: 320mg  D4-6: 400mg  D7:480mg | 17 | | Double blind randomised placebo control trial | | DSM III bipolar disorder: acute mania or mixed episode  Hospitalised cohort | | MRS | | 21 days | | No difference between groups | | Not reported | |
| Mallinger 2008 [5] | P | Verapamil  D1: 160mg  D4:240mg  D7 : 320mg  D10: 400mg  D13: 480mg | 10 | | Double blind randomised control trial  Lithium comparator | | DSM IV bipolar disorder or schizoaffective disorder: acute mania  Lithium non responders | | Raskin severity of mania scale  BRMS | | 21 days | | Verapamil showed minimal  efficacy for treating manic patients who previously  failed to respond to an initial three-week trial of  lithium | | Not reported | |
| Pal Singh 2008 [6] | P | Verapamil  D1: 160mg  D2: 240mg  Adjusted up to 320mg/day thereafter – “flexibly dosed” | 25 | | Double blind randomised control trial  Lithium comparator | | ICD-10 Mania  Hospitalised cohort | | BRMS  YMRS | | 28 days | |  | | Akathisia (N=1)  Tremors (N=2)  Rigidity (N=1)  Salivation (N=2)  Sedation (N=2)  Constipation (N=4)  Glossitis (N=1) | |
| **Non-randomised blinded trials** | | | | | | | | | | | | | | | |  |
| Aldenhoff 1986 [7] | P | D600  75mg | 10 | | Double blind placebo controlled trial. Non randomised | | DSM III Mania  Hospitalised cohort | | GAS  BRMS  IMPS | | 15 days | | Statistically significant improvement in IMPS motor disturbance subscale | | One participant required additional psychotropic medication for uncontrolled aggressiveness. | |
| Dose 1986 [8] | P | Verapamil  320-480mg | 8 | | Double blind A-B-A placebo controlled study | | Manic or schizomanic syndrome | | IMPS | | 7 days | | Average reduction in IMPS of 30% | | “Slight changes in blood pressure, heart rates and digestion” | |
| Giannini 1985 [9] | P | Verapamil 80mg qds | 20 | | Double blind crossover study  Clonidine comparator | | DSM III mania | | BPRS | | 20 days | | Verapamil  superior to clonidine on days 10 (U = 25, P < .05)  and day 20 (U = 8, P < .001). | | No adverse events | |
| Hoschl 1989 [10] | P | Verapamil  480mg | 12 | | Controlled double blind study  Other psychotropic comparators | | DSM III mania  Hospitalised cohort | | HAMD  BPRS | | 35 days | | Improvement in BPRS comparable with standard treatments | | No adverse events | |
| Nurnberger 1987 [11] |  | Diltiazem 5-15mg iv over 10 minutes | 2 | | Single blind study | | Euthymic bipolar disorder | | Not reported | | Not reported | | No mood effects noted | | Not reported | |
| Pazzaglia 1998 [12] | D | Nimodipine  360mg or maximum tolerate dose | 23 | | Double blind study  Monotherapy followed by Carbamazepine augmentation | | Monotherapy:  Bipolar I disorder (N=7)  Bipolar II disorder (N=16)  Augmentation:  Bipolar I disorder (N=5)  Bipolar II disorder (N=6) | | CGI | | Variable | | 12 responded to monotherapy  3/11 non responders responded to addition of carbamazepine.  Response defined as moderate or marked improvement on combination of mood and CGI measure. | | Not reported | |
| **Randomised single-blinded trials** | | | | | | | | | | | | | | | |  |
| Walton 1996 [13] | P | Verapamil  Dose not reported | 40 | | Single blind randomised controlled trial  Lithium comparator | | DSM IV mania | | BPRS  MRS  GAF  CGI | | 28 days | | Lithium showed significant improvements in the BPRS, MRS, GAF and CGI compared with verapamil | | Not reported | |
| **Observational studies** | | | | | | | | | | | | | | | |  |
| Barton 1987 [14] | P | Verapamil  Acute mania 80mg tds  Recurrent episodes 240-320mg  Pharmacological mania 80mg tds – 80mgs qds | 14 | | Open label study  Adjunctive treatment | | RDC bipolar-I disorder (N=10), bipolar-II disorder (N=3) and schizoaffective disorder (N=1)  Acute mania (N=8)  Recurrent bipolar episodes (N=4)  Pharmacological mania (N=2)  Community sample | | YMRS | | Acute mania: 21 days  Maintenance: 18 months  Pharmacological mania: 14 days | | No improvement in acute mania  2 had mild improvement in recurrence of episodes  In those with pharmacological mania Verapamil prevented antidepressant induced mania | | Dysphoric feelings  Headache  Constipation | |
| Brotman 1986 [15] | P | Verapamil  80mg tds | 6 | | Open label study  Adjunctive treatment | | DSM III bipolar disorder  Acute mania  Hospitalised | | BPRS  YMRS | | Not reported | | All patients had reductions in their BPRS and YMRS scores. | | No adverse events | |
| Brunet 1990 [16] | D | Nimodipine  360mg 4 hourly | 6 | | Open label study | | DSM IIIR bipolar disorder  Acute mania  Hospitalised | | BRMS  BPRS | | 7 days | | Significant improvements in Bech scale (p<0.001) and BPRS (p<0.02) | | Sleep disturbance (may be due to might time administration of the drug) | |
| Caillard 1985 [17] | B | Diltiazem  120mg-360mg | 5 | | Open label study | | DSM III Mania  Hospitalised | | MSRS | | 14 days | | Significant improvements in MSRS >70% in all patients | | Headache (N=1)  Vertigo without orthostatic hypotension (N=1)  Transient oedema of extremities (N=1) | |
| Dinan 1988 [18] | P | Verapamil  400mg | 6 | | Open label study  Adjunctive temazapem | | DSM III mania  Hospitalised | | Petterson mania scale | | 21 days | | 5/6 improved significantly on the Petterson mania scale by 14 days. 3/5 relapsed by 21 days | | Not reported | |
| Giannini 1984 [19] | P | Verapamil  80mg qds | 12 | | Repeated measures design  Monotherapy  Lithium comparator | | DSM III mania | | BPRS | | 30 days | | Verapamil equally effective to lithium in terms of change in BPRS from D0 to D30 | | Not reported | |
| Goodnick 1996 [20] | P | Verapamil  120mg M/R mane  240mg M/R nocte | 12 | | Open label study | | DSM IIIR mania | | YMRS | | 14 days | | Improvement in YMRS scores of 60.5%.  Improvement in mania correlated significantly with increased plasma calcium (p=0.034) | | Not reported | |
| Hoschl 1985 [21] | P | Verapamil | 5 | | Open label study | | Mania  Hospitalised | | BPRS | | 14 days | | All patients reached full remission | | Not reported | |
| Lenzi 1995 [22] | P | Verapamil  400mg | 15 | | Open label study  Adjunctive chlorpromazine permitted | | DSM IIIR mania  Hospitalised cohort | | BPRS  CGI | | 21 days  (Improvements in BPRS across the group by day 10). | | 1 patient responded to verapamil monotherapy.  2 required night time chlorpromazine. 12 required higher doses of chlorpromazine | | Sweating (N=8)  Dizziness (N=2)  Tachycardia (N=5)  Constipation (N=5)  Blurred vision (N=5)  Hypotension (N=1) | |
| Manna 1991 [23] | D | Nimodipine 30mg tds | 12 | | Open label study  Monotherapy versus lithium & nimodipine | | DSM III rapid cycling bipolar disorder | | BPRS | | 6 months | | Association of lithium and nimodipine more effective than lithium or nimodipine monotherapy | | Not reported | |
| Ostacher 2014 [24] | D | Isradipine  5mg bd | 12 | | Open label study  Adjunctive treatment | | DSM IV bipolar I disorder (N=  DSM IV bipolar II disorder (N=  Acute depression | | MADRS  QUIDS-SR  CGI-S  YMRS | | 8 weeks | | Mean MADRS decrease of 2.1 /wk. Mean QUIDS-SR decrease of 1.0/week. Mean CGI-S decrease of 0.18/wk.  No significant change in YMRS noted. | | Initial decrease in diastolic blood pressure and increased heart rate. Both returned to normal at 4 weeks. | |
| Pazzaglia 1995 [25] | D | Nimodipine | 10 | | Open label study | | Rapid cycling bipolar disorder  Hospitalised | | Bunney-Hamburg rating scales for mania, depression, anger, psychosis and anxiety | | 26-98 days | | Depressive symptoms worsened in 5/10, 2/10 had minor improvements, 3/10 no mood change | | Not reported | |
| Silverstone 2000 [26] | B | Diltiazem  60mg bd – 120mg bd | 8 | | Retrospective analysis of clinical outcomes  Adjunctive treatment | | DSM IV bipolar disorder  Community sample | | DSM IV diagnostic criteria | | 6 months | | Significant treatment between pre and post treatment symptom scores (F=88.2. p<0.0001) | | Nausea (N=2)  Headache (N=1) | |
| Snedkova  1997 [27] | D | Nifedipine | 26 | | Open label study  Lithium comparator | | ICD 10.  Affective and schizoaffective psychoses | | Number of mood episodes.  Number of hospital admissions | | 2 years | | 46.8% reduction in duration of mood episodes  31.7% reduction in frequency of mood episodes  52.2% reduction in hospital admissions. | | Dry mouth (N=4)  Peripheral vasodilation (N=3)  Iridocyclitis (N=1)  Headaches (N=4)  Digestive problems (N=4)  Sleep disturbance (N=4)  Leg oedema (N=1) | |
| **Case reports/Case series** | | | | | | | | | | | | | | | |  |
| Davanzo 1999 [28] | D | Nimodipine  60mg tds | | 1 | | Case report  13 yr old male  Adjunctive treatment | Bipolar 1 disorder  Ultradian cycling treatment resistant  Acute mania | Not reported | | 3 years | | Symptoms started to improve at day 6. In remission to follow-up  On attempted reduction of nimodipine hypomania emerged. | | No adverse events | |  |
| Deicken 1990 [29] | P | Verapamil  320mg in divided doses | | 1 | | Case report  44 yr old male  Treatment resistant Adjunctive treatment | DSM IIIR bipolar disorder  Acute depression | BDI  HAMD | | 9 months | | No further depressive episodes during the follow-up period. | | Intermittent mild headaches | |  |
| Dubovsky 1982 [30] | P | Verapamil  80mg bd | | 1 | | Case report  53yr old female | Acute mania | MRS | | 21 days | | Significant improvement in symptoms from day 3. Withdrawal of verapamil associated with re-emergence of manic symptoms | | No adverse events | |  |
| Dubovsky 1985 [31] | P | Verapamil  400mg | | 1 | | Case report  48yr old woman currently depressed with history of antidepressant induced mania. | Bipolar disorder.  Antidepressant induced mania | MSRS  BPRS | | 6 weeks | | Verapamil had no impact upon depressive symptoms. On the introduction of an antidepressant mood improved with no manic symptoms. Withdrawal of Verapamil was followed by manic episode.  Re-introduction resolved mania and she remained symptom free for 8 months. | | Not reported | |  |
| Dubovsky 1987 [32] | P | Verapamil  P1. 480mg  P2. 320mg | | 2 | | Case reports  Adjunctive treatment | Bipolar disorder  P1. Rapid cycling  P2. Acute mania | Clinical impression | | 21 days | | P1. No improvement  P2. Mania improved | | P1. Sinus bradycardia  P2. Sinus bradycardia with class 1 atrioventricular ectopy. Died of MI 3 days later | |  |
| Gitlin 1984 [33] | P | Verapamil  80mg tds | | 1 | | Case report  32 yr old woman  Adjunctive treatment | Bipolar disorder  Treatment resistant | BDI  BPRS | | 15months | | Euthymic except 4 brief periods of hypomania from commencing verapamil | | Headaches  Constipation | |  |
| Goodnick 1993 [34] | P | Verapamil  180mg or 240mg M/R | | 3 | | Case series  Pregnant women  Monotherapy | Bipolar disorder  Acute mania( N=2)  Relapse prevention (N=1) | Not reported | | Not reported | | Remission in all 3 women throughout the pregnancy | | Not reported | |  |
| Goodnick 1995 [35] | D | Nimodipine  60mg tds | | 2 | | Case series  P1. 53 yr old woman  P2. 58 yr old man | Rapid cycling bipolar disorder | Not reported | | P1. 12 months  P2. 5 months | | Mood stability maintained | | Not reported | |  |
| Grunze 1996 [36] | P | Nimodipine  270mg/d | | 1 | | Case report  42 yr old female  Adjunctive treatment | DSM IV mania | BPRS, HAMD, BRMAS | | 42 days | | Improvement in BPRS, HAMD, BRMAS  No observed interaction with Lithium  2 months after discontinuing Nimodipine the patient developed acute mania | | No adverse events | |  |
| Helmuth 1989 [37] | P | Verapamil  120mg | | 1 | | Case report  Adjunctive treatment | DSM IIIR mania | Not reported | | 4 days | | Manic symptoms resolved following addition of verapamil.  Symptoms re-emerged on withdrawal | | Involuntary choreoathetoid movements.  Resolved on withdrawal of verapamil. | |  |
| Hesslinger 1996 [38] | D | Nimodipine 360mg | | 1 | | Case report  56 yr old female treatment resistant | Bipolar disorder current episode depressed | HAMD | | Not stated | | Remission by 26 days and euthymic for nearly a year | | Not reported | |  |
| Jacobsen 1987 [39] | P | Verapamil  80mg qds reduced to bd on reintroduction. | | 1 | | Case report  24 yr old female | Rapid cycling bipolar disorder | Clinical impression | | 7 days | | No improvement in mood cycling until the introduction of amitriptyline 25mg and decrease in verapamil to 160mg. | | Toxic delirium at 7 days. Symptoms re-emerged following discontinuation and reinstitution of verapamil | |  |
| Kennedy 1986 [40] | P | Verapamil | | 2 | | Case reports  P1: 38 yr old female  P2: 32 yo male  Monotherapy with oxazepam as a hypnotic | Refractory bipolar disorder | MMRS | | 30 days | | No response noted in either patient | | Not reported | |  |
| Mathis 1988 [41] | P | Verapamil 320mg | | 7 | | Case series | DSM III mania  Bipolar disorder (N=4)  Schizoaffective (N=3) | Not reported | | 28 days | | 4/7 improved | | 3 subsequently had a depressive relapse between the 12^th^ and 15^th^ day of treatment. | |  |
| Patterson 1987 [42] | P | Verapamil  80mg qds | | 1 | | Case report  68yr old male  Monotherapy | Acute mania | Not reported | | 6 months | | No recurrence of mood episodes at 6 month follow-up | | Not reported | |  |
| Price 1986 [43] | P | Verapamil  80mg tds | | 1 | | Case report  54 yr old woman  Adjunct to carbamazepine | Manic depressive illness | Clinical impression | | 7 days | | None reported | | Persistent nausea, ataxia and incoordination. Mild memory impairment. All resolved on withdrawal of verapamil | |  |
| Price 1988 [44] | P | Verapamil  80mg tds | | 1 | | Case report  42 yr old woman  Adjunct to lithium | Rapid cycling bipolar disorder | Clinical impression | | 9 days | | At 7 days no longer exhibited pressured speech, insomnia or grandiosity | | Nausea, vomiting, muscular weakness,  ataxia, and “ringing of the ears.” Physical examination  revealed an ataxic gait. Compete resolution following withdrawal of verapamil. Re-challenge with verapamil provoked re-emergence of side effects. | |  |
| Russova 1998 [45] | P | Verapamil | | 4 | | Case series  Adjunctive treatment | DSM III mania | BPRS, CGI | | 21 days | |  | |  | |  |
| Solomon 1986 [46] | P | Verapamil  P1: 80mg qds  P2: 8-mg tds | | 2 | | Case report  P1: 51 yr old female  P2: 71 yr old male  Adjunctive antidepressant treatment | Bipolar disorder  Lithium responders in whom lithium had become contraindicated. | Not reported | | Not reported | | Euthymia maintained at 4 months | | No adverse events | |  |
| Wisner 2002 [47] | P | Verapamil  480mg maximum | | 37 | | Case series  Monotherapy | DSM IV bipolar disorder  Acute mania/hypomania (N=2)  Mixed affective state (N=9)  Acute depression (N=18)  Euthymic (N=9)  Pregnant women (N=29) | HAMD  MRS BRMS  IDD | | Minimum 1 month | | 2/2 with mania responded  7/9 with mixed states responded (7 had resolution of manic symptoms, 2 of depressive symptoms)  7/18 with depression responded  6/9 euthymic women remained well | | Headache (N=1)  Rash (N=1)  Palpitations (N=1) | |  |

**Legend.** Abbreviations: P= Phenilalkylamines; B= Benzothiazepines; D= Dihydropyridines; BDI – Beck Depression inventory; BPRS – Brief psychiatric rating scale; BMMRS – Beigel and Murphy mania rating scale; BRMS - Bech-rafaelsen mania rating scale; CGI – Clinical Global Index of severity; GAF – Global assessment of function; GAS - Global assessment scale; HAMD - Hamilton depression rating scale; IDD – Inventory to diagnose depression; IMPS – Inpatient-multidimensional psychiatric scale; MADRS - Montgomery-Asberg depression rating scale; MRS – Mania rating scale; MSRS - Manic state rating scale; QUIDS-SR - Quick inventory of depression score self-report; YMRS – Young mania rating scale; tds: three times per day; qds: four times a day; bd: twice a day.

**References to included studies**

1. Dubovsky, S.L., et al. *Calcium antagonists in mania: a double-blind study of verapamil*. Psychiatry research, 1986. **18**, 309-20.

2. Garza-Trevino ES, Overall JE, Hollister LE. *Verapamil versus lithium in acute mania.* American Journal of Psychiatry, 1992. **149**(1): p. 121-122.

3. Giannini, A.J., R. Taraszewski, and R.H. Loiselle *Verapamil and lithium in maintenance therapy of manic patients*. Journal of clinical pharmacology, 1987. **27**, 980-2.

4. Janicak, P.G., et al. *Verapamil for the treatment of acute mania: a double-blind, placebo-controlled trial*. American journal of psychiatry, 1998. **155**, 972-3.

5. Mallinger, A.G., et al. *Verapamil augmentation of lithium treatment improves outcome in mania unresponsive to lithium alone: preliminary findings and a discussion of therapeutic mechanisms*. Bipolar disorders, 2008. **10**, 856-66 DOI: 10.1111/j.1399-5618.2008.00636.x.

6. Pal Singh, G. *A double-blind comparative study of clinical efficacy of verapamil versus lithium in acute mania*. International journal of psychiatry in clinical practice, 2008. **12**, 303-8.

7. Aldenhoff, J.B., et al. *Antimanic effects of the calcium-antagonist D600. A double-blind placebo-controlled study*. Clinical neuropharmacology, 1986. **9 Suppl 4**, 553-5.

8. Dose, M., et al., *Use of calcium antagonists in mania.* Psychoneuroendocrinology, 1986. **11**(2): p. 241-243.

9. Giannini, A.J., et al. *Comparison of antimanic efficacy of clonidine and verapamil*. Journal of clinical pharmacology, 1985. **25**, 307-8.

10. Höschl, C. and J. Kozený *Verapamil in affective disorders: a controlled, double-blind study*. Biological psychiatry, 1989. **25**, 128-40.

11. Nurnberger, J. and S. Simmons-Alling, *Mediation of "calcium antagonist" effects by dopamine receptor blockade.* The American Journal of Psychiatry, 1987. **144**(7): p. 966-967.

12. Pazzaglia, P.J., et al. *Nimodipine monotherapy and carbamazepine augmentation in patients with refractory recurrent affective illness*. Journal of clinical psychopharmacology, 1998. **18**, 404-13.

13. Walton, S.A., M. Berk, and S. Brook *Superiority of lithium over verapamil in mania: a randomized, controlled, single-blind trial*. Journal of clinical psychiatry, 1996. **57**, 543-6.

14. Barton BM, Gitlin MJ. *Verapamil in treatment-resistant mania: an open trial.* Journal of Clinical Psychopharmacology, 1987. **7**(2): p. 101-3.

15. Brotman AW, Farhadi AM, Gelenberg AJ. *Verapamil treatment of acute mania.* Journal of Clinical Psychiatry, 1986. **47**(3): p. 136-138.

16. Brunet, G., et al., *Open trial of a calcium antagonist, nimodipine, in acute mania.* Clinical Neuropharmacology, 1990. **13**(3): p. 224-228.

17. Caillard, V., *Treatment of mania using a calcium antagonist - Preliminary trial.* Neuropsychobiology, 1985. **14**(1): p. 23-26.

18. Dinan, T.G., T. Silverstone, and J.C. Cookson, *Cortisol, prolactin and growth hormone levels with clinical ratings in manic patients treated with verapamil.* International clinical psychopharmacology, 1988. **3**(2): p. 151-156.

19. Giannini, A.J., W.L. Houser Jr, and R.H. Loiselle, *Antimanic effects of verapamil.* American Journal of Psychiatry, 1984. **141**(12): p. 1602-1603.

20. Goodnick, P.J., *Treatment of mania: Relationship between response to verapamil and changes in plasma calcium and magnesium levels.* Southern Medical Journal, 1996. **89**(2): p. 225-226.

21. Hoschl, C., et al., *Experience with verapamil (Isoptin Knoll) in psychiatry.* Activitas Nervosa Superior, 1985. **27**(4): p. 253-255.

22. Lenzi, A., et al., *Effectiveness of the combination verapamil and chlorpromazine in the treatment of severe manic or mixed patients.* Progress in Neuro-Psychopharmacology and Biological Psychiatry, 1995. **19**(3): p. 519-528.

23. Manna, V., *Bipolar affective disorder and intraneuronal calcium. Treatment with lithium salts and/or calcium antagonist in patients with rapid polar inversion of mood tone. [Italian] Disturbi Affettivi Bipolari E Ruolo Del Calcio Intraneuronale. Effetti Terapeutici Del Trattamento Con Sali Di Litio E/O Calcio Antagonista in Pazienti Con Rapida Inversione Di Polarita.* Minerva Medica, 1991. **82**(11): p. 757-763.

24. Ostacher, M.J., et al., *Pilot investigation of isradipine in the treatment of bipolar depression motivated by genome-wide association.* Bipolar Disorders, 2014. **16**(2): p. 199-203.

25. Pazzaglia, P.J., et al., *Nimodipine increases CSF somatostatin in affectively ill patients.* Neuropsychopharmacology, 1995. **13**(1): p. 75-83.

26. Silverstone, P.H. and L. Birkett, *Diltiazem as augmentation therapy in patients with treatment-resistant bipolar disorder: A retrospective study.* Journal of Psychiatry and Neuroscience, 2000. **25**(3): p. 276-280.

27. Snedkova, L.V., et al., *The use of nifedipine for overcoming the insufficient preventive effect of lithium carbonate in patients with affective and schizoaffective psychoses. [Russian]Ispol'zovanie nifedipina dlia preodoleniia nedostatochnogo preventivnogo effekta karbonata litiia u bol'nykh affektivnymi i shizoaffektivnymi psikhozami.* Zhurnal nevrologii i psikhiatrii imeni S.S, 1997. Korsakova / Ministerstvo zdravookhraneniia i meditsinskoi promyshlennosti Rossiiskoi Federatsii, Vserossiiskoe obshchestvo nevrologov [i] Vserossiiskoe obshchestvo psikhiatrov. 97(10): p. 35-39.

28. Davanzo, P.A., et al., *Nimodipine treatment of an adolescent with ultradian cycling bipolar affective illness.* Journal of Child and Adolescent Psychopharmacology, 1999. **9**(1): p. 51-61.

29. Deicken, R.F., *Verapamil treatment of bipolar depression.* Journal of Clinical Psychopharmacology, 1990. **10**(2): p. 148-149.

30. Dubovsky SL et al., *Effectiveness of verapamil in the treatment of a manic patient.* American Journal of Psychiatry, 1982. **139**(4): p. 502-504.

31. Dubovsky SL, RD Franks,Schrier D. *Phenelzine-induced hypomania: Effect of verapamil.* Biological Psychiatry, 1985. **20**(9): p. 1009-1014.

32. Dubovsky, S.L., R.D. Franks, and S. Allen, *Verapamil: A new antimanic drug with potential interactions with lithium.* Journal of Clinical Psychiatry, 1987. **48**(9): p. 371-372.

33. Gitlin, M.J. and J. Weiss, *Verapamil as maintenance treatment in bipolar illness: A case report.* Journal of Clinical Psychopharmacology, 1984. **4**(6): p. 341-343.

34. Goodnick, P.J., *Verapamil prophylaxis in pregnant women with bipolar disorder.* The American Journal of Psychiatry, 1993. **150**(10): p. 1560.

35. Goodnick, P.J., *Nimodipine treatment of rapid cycling bipolar disorder.* Journal of Clinical Psychiatry, 1995. **56**(7): p. 330.

36. Grunze, H., et al., *Combined treatment with lithium and nimodipine in a bipolar I manic syndrome.* Progress in Neuro-Psychopharmacology and Biological Psychiatry, 1996. **20**(3): p. 419-426.

37. Helmuth D et al., *Choreoathetosis induced by verapamil and lithium treatment.* Journal of Clinical Psychopharmacology, 1989. **9**(6): p. 454-5.

38. Hesslinger, B., et al., *Nimodipine in the prophylaxis of bipolar affective disorder. A case report. [German] Phasenprophylaxe Bei Bipolar Affektiver Storung Mit Nimodipin. Ein Fallbericht.* Nervenarzt, 1996. **67**(5): p. 394-396.

39. Jacobsen, F.M., D.A. Sack, and S.P. James, *Delirium induced by verapamil.* The American Journal of Psychiatry, 1987. **144**(2): p. 248.

40. Kennedy, S., S. Ozersky, and M. Robillard, *Refractory bipolar illness may not respond to verapamil.* Journal of clinical psychopharmacology, 1986. **6**(5): p. 316-317.

41. Mathis, P., L. Schmitt, and P. Moron, *Effectiveness of verapamil in acute mania. [French] Efficacite Du Verapamil Dans Les Acces Maniaques.* Encephale, 1988. **14**(3): p. 127-132.

42. Patterson, J.F., *Treatment of acute mania with verapamil.* Journal of clinical psychopharmacology, 1987. **7**(3): p. 206-207.

43. Price, W.A. and A.J. Giannini, *Neurotoxicity caused by lithium-verapamil synergism.* Journal of Clinical Pharmacology, 1986. **26**(8): p. 717-9.

44. Price, W.A. and L.R. DiMarzio, *Verapamil-carbamazepine neurotoxicity.* The Journal of clinical psychiatry, 1988. **49**(2): p. 80.

45. Russova, A., et al., *A calcium antagonist (verapamil) in treatment of severe episodes of mania. [Italian].* Rassegna di Studi Psichiatrici, 1988. **77**(1-2): p. 264-269.

46. Solomon, L. and P. Williamson, *Verapamil in bipolar illness.* The Canadian Journal of Psychiatry / La Revue canadienne de psychiatrie, 1986. **31**(5): p. 442-444.

47. Wisner, K.L., et al., *Verapamil treatment for women with bipolar disorder.* Biological Psychiatry, 2002. **51**(9): p. 745-752.
